# Practical considerations of diffusion-weighted MRS with ultra-strong diffusion gradients

**DOI:** 10.3389/fnins.2023.1258408

**Published:** 2023-12-07

**Authors:** Christopher W. Davies-Jenkins, André Döring, Fabrizio Fasano, Elena Kleban, Lars Mueller, C. John Evans, Maryam Afzali, Derek K. Jones, Itamar Ronen, Francesca Branzoli, Chantal M. W. Tax

**Affiliations:** ^1^The Russell H. Morgan Department of Radiology and Radiological Science, Johns Hopkins University School of Medicine, Baltimore, MD, United States; ^2^Kirby Research Center for Functional Brain Imaging, Kennedy Krieger Institute, Baltimore, MD, United States; ^3^Cardiff University Brain Research Imaging Centre, Cardiff University, Cardiff, United Kingdom; ^4^CIBM Center for Biomedical Imaging, EPFL CIBM-AIT, EPFL Lausanne, Lausanne, Switzerland; ^5^Siemens Healthcare Ltd., Camberly, United Kingdom; ^6^Department of Radiology, Universität Bern, Bern, Switzerland; ^7^Leeds Institute of Cardiovascular & Metabolic Medicine, University of Leeds, Leeds, United Kingdom; ^8^Clinical Sciences Institue, Brighton and Sussex Medical School, Brighton, United Kingdom; ^9^Center for NeuroImaging Research (CENIR), Paris Brain Institute (ICM), Pitié-Salpêtrière Hospital, Paris, France; ^10^Inserm U1127, CNRS U7225, Sorbonne Universités, Paris, France; ^11^Brain Research Imaging Centre, School Physics and Astronomy, Cardiff University, Cardiff, United Kingdom; ^12^Image Sciences Institute, University Medical Center Utrecht, Utrecht, Netherlands

**Keywords:** diffusion-weighted MRS, ultra-strong gradients, gradient non-uniformity, eddy currents, metabolites

## Abstract

**Introduction:**

Diffusion-weighted magnetic resonance spectroscopy (DW-MRS) offers improved cellular specificity to microstructure—compared to water-based methods alone—but spatial resolution and SNR is severely reduced and slow-diffusing metabolites necessitate higher *b*-values to accurately characterize their diffusion properties. Ultra-strong gradients allow access to higher *b*-values per-unit time, higher SNR for a given *b*-value, and shorter diffusion times, but introduce additional challenges such as eddy-current artefacts, gradient non-uniformity, and mechanical vibrations.

**Methods:**

In this work, we present initial DW-MRS data acquired on a 3T Siemens Connectom scanner equipped with ultra-strong (300 mT/m) gradients. We explore the practical issues associated with this manner of acquisition, the steps that may be taken to mitigate their impact on the data, and the potential benefits of ultra-strong gradients for DW-MRS. An in-house DW-PRESS sequence and data processing pipeline were developed to mitigate the impact of these confounds. The interaction of TE, *b*-value, and maximum gradient amplitude was investigated using simulations and pilot data, whereby maximum gradient amplitude was restricted. Furthermore, two DW-MRS voxels in grey and white matter were acquired using ultra-strong gradients and high *b*-values.

**Results:**

Simulations suggest T_2_-based SNR gains that are experimentally confirmed. Ultra-strong gradient acquisitions exhibit similar artefact profiles to those of lower gradient amplitude, suggesting adequate performance of artefact mitigation strategies. Gradient field non-uniformity influenced ADC estimates by up to 4% when left uncorrected. ADC and Kurtosis estimates for tNAA, tCho, and tCr align with previously published literature.

**Discussion:**

In conclusion, we successfully implemented acquisition and data processing strategies for ultra-strong gradient DW-MRS and results indicate that confounding effects of the strong gradient system can be ameliorated, while achieving shorter diffusion times and improved metabolite SNR.

## 1 Introduction

Diffusion-weighted magnetic resonance imaging (DW-MRI) is usually sensitized to the displacement of water, and provides a myriad of tissue microstructure metrics that aid in the study of many neuropathologies, including traumatic brain injury (Hutchinson et al., 2018), neurodegeneration (Goveas et al., 2015), and measuring treatment response in cancer therapy (Patterson et al., 2008), to name a few. However, the ubiquity of water molecules—present in both intra- and extracellular spaces—complicates the modeling of water diffusion as a measure of cellular microstructure. Magnetic resonance spectroscopy (MRS) is a non-invasive technique providing quantitative measures of metabolites and neurotransmitters which are present in the brain at millimolar concentrations. Diffusion-weighted MRS (DW-MRS) introduces diffusion gradients into MRS sequences, utilising MRS as a filter to sensitize the MR signal to different metabolites which are almost exclusively intra-cellular, with some considered predominantly glial—myo-inositol (mI) and choline compounds (tCho)—and others predominantly neuronal—N-acetyl-aspartate (NAA) and glutamate (Glu) (Choi et al., 2007). While the signal-to-noise ratio (SNR) and spatial resolution are reduced compared to conventional water-based imaging, the specificity afforded by DW-MRS greatly simplifies diffusion modeling and interpretation, and provides a valuable non-invasive window into metabolism and cellular microstructure, complimentary to water-based diffusion imaging (Ronen et al., 2014; Najac et al., 2016; Palombo et al., 2018; Ligneul et al., 2019; Genovese et al., 2021b).

The apparent diffusion coefficients (ADCs) of metabolites are at least five times smaller than those of water (Ellegood et al., 2011), which necessitates higher *b*-values to adequately characterize metabolite diffusion properties. A common approach is to employ a DW-STEAM sequence, whereby metabolite diffusion occurs during the mixing time (TM), with the diffusion time uncoupled from the echo time (TE). However, this comes with the caveat that STEAM generates a stimulated echo, reducing the available SNR compared to spin-echo localisation methods by a factor of two. The shorter TE of STEAM can ameliorate this, but the long diffusion times required to achieve adequate diffusion weighting may be undesired. If the goal is to probe short diffusion times and/or high *b*-values, then the spin-echo-based diffusion-weighted Point RESolved Spectroscopy sequence (Bottomley, 1987) (DW-PRESS) provides an alternative, offering better SNR than STEAM, without the additional TE restrictions imposed by adiabatic pulse pairing required for a diffusion-weighted semi-LASER (DW-sLASER) sequence. Larger *b*-values can be achieved by increasing the DW-gradient amplitude (limited by the gradient system) and/or by increasing the diffusion time. Achieving the latter with DW-PRESS can be challenging. With the diffusion time coupled to the choice of TE, the available SNR at high *b*-value is restricted by metabolite *T*_2_ relaxation. The introduction of ultra-strong gradient systems can mitigate this. The Siemens Connectom scanner is fitted with a gradient system capable of reaching 300 mT/m per axis. This provides larger *b*-values for a given TE, and access to shorter diffusion times while maintaining the required *b*-value range. Shorter diffusion times can reduce the variability resulting from motion artefacts and, crucially, can provide additional cell-specific microstructural properties of highly-restrictive compartments (e.g., subcellular organelles) and cellular viscosity (Setsompop et al., 2013; Palombo et al., 2017; Jones et al., 2018).

However, the introduction of ultra-strong gradients poses additional practical challenges. Specifically, eddy currents become increasingly prevalent at larger gradient amplitudes. Switched gradient fields induce eddy currents which produce time-varying magnetic fields, distorting the lineshape of MR spectra and hampering MRS modeling attempts. Moreover, eddy current correction in conventional MRS relies on acquiring a water-unsuppressed reference scan (Klose, 1990); however, in DW-MRS, the water signal is heavily attenuated at high *b*-values, complicating the extraction of the relevant phase information. In addition to eddy current effects, it becomes increasingly difficult to maintain uniform gradient fields on ultra-strong gradient systems. This leads to a deviation in the applied gradient field from the nominal gradient field, which is of particular relevance for diffusion studies (Mesri et al., 2020). These gradient non-uniformities will spatially modulate the *b*-matrix and image geometry, and must be corrected in order to obtain reliable estimates (Bammer et al., 2003). Finally, mechanical vibrations—caused by Lorentz forces generated during rapid gradient switching—can cause anomalous signal loss in diffusion experiments (Gallichan et al., 2010; Weidlich et al., 2019, 2020), and is a particular concern for strong gradient systems.

At the time of writing, only one full study has been published on DW-MRS with ultra-strong gradients in humans, which focused on utilising the hardware to measure macromolecular background profiles for MRS (Şimşek et al., 2022). Despite increasing community interest in strong gradients for diffusion encoding (Setsompop et al., 2013; Jones et al., 2018; Jenkins et al., 2020; Şimşek et al., 2020, 2021; Huang et al., 2021; Jenkins, 2021; Fan et al., 2022; Döring et al., 2023), no studies have specifically addressed the challenges of ultra-strong gradients in the context of DW-MRS. Furthermore, data processing software specific to DW-MRS data is limited, and variation between existing methods has been previously shown (Najac et al., 2022). With increased accessibility of high performance scanner gradient systems, it is crucial to develop open-source software that broaches the challenges posed by this hardware.

In this work, we present initial data acquired using a 3T Connectom—a research-only scanner equipped with a 300 mT/m gradient system. We introduce the practical issues associated with this manner of acquisition and steps that may be taken to mitigate their impact on the data. In this study, we limited the scope of our investigation specifically to the diffusion-weighted PRESS sequence, which was implemented with a flexible bipolar diffusion gradient scheme. A DW-MRS data processing pipeline was implemented and evaluated against pilot DW-PRESS data. Phantom experiments were conducted and *in vivo* data were acquired from a small cohort of individuals in order to validate the measurements and demonstrate the capabilities of ultra-strong gradients compared to more conventional gradient settings.

## 2 Materials and methods

**Figure 1:**
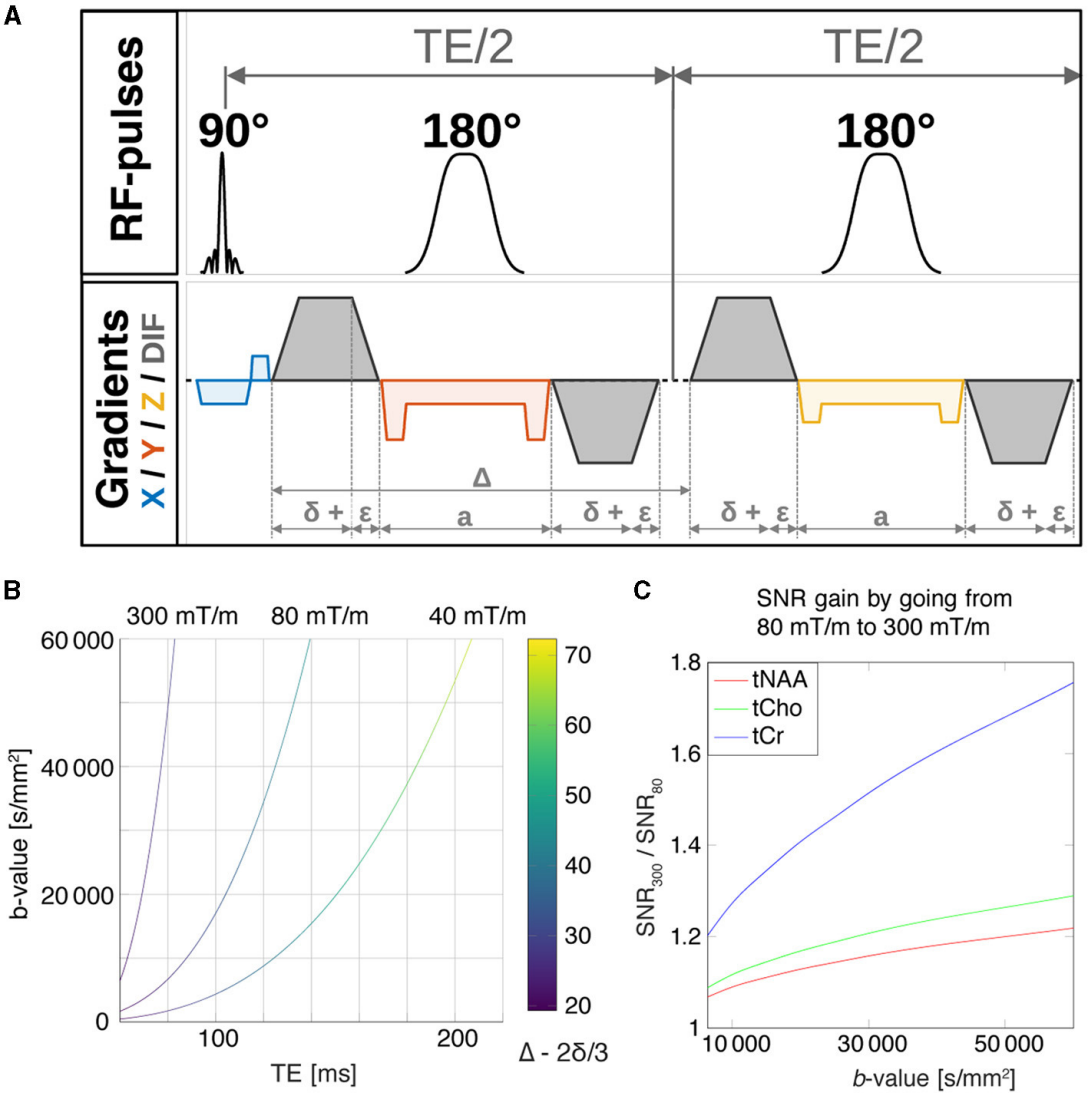
**(A)** Pulse sequence diagram for the DW-PRESS sequence. Slice-select gradients are shown for each dimension, x (blue), y (red), z (yellow), and diffusion gradients are shown in gray. **(B)** The maximum achievable *b*-value as a function of the echo time for the DW-PRESS sequence, which is limited by the maximum achievable diffusion-gradient strengths. The curves were calculated for maximal gradient amplitudes of 40, 80, and 300 mT/m corresponding to the Siemens TRIO, Prisma, and Connectom scanner configurations, respectively. **(C)** For a given *b*-value, reduction of the echo time by going from 80 mT/m to 300 mT/m will result in SNR gain. SNR gains were estimated for tNAA, tCho and tCr using their relaxation times *T*_2_ (Ke et al., 2002; Ganji et al., 2012).

For the DW-MRS acquisition, an in-house developed DW-PRESS sequence with bipolar diffusion gradients (Branzoli, 2015) was adapted for use on the 300 mT/m Connectom scanner. The full pulse sequence diagram is shown in Figure 1A.

### 2.1 SNR simulations

We first conducted simulations to explore the relationship between TE, maximum *b*-value, and diffusion time for the DW-PRESS sequence, and subsequently, the potential TE-based SNR improvements possible with ultra-strong gradients. The maximum-achievable *b*-value is constrained by the gradient characteristics, and has a corresponding minimum-achievable TE. We investigated this relationship at three maximum gradient amplitudes: 40, 80, and 300 mT/m. The maximum achievable *b*-value was calculated numerically using in-house code written in Matlab. The slew rates—corresponding to each of the maximum gradient amplitudes—were 200 T/m/s, 200 T/m/s and 83 T/m/s, respectively.

For a given maximum *b*-value, the minimum-achievable TE will define the magnitude of *T*_2_-based signal attenuation, with short TE implying higher SNR. We investigated the expected SNR increase when moving from a Siemens Prisma gradient system (80mT/m) to a Connectom gradient system (300mT/m) for three major metabolite measures—creatine + phosphocreatine (tCr), tCho, and NAA+N-acetyl-aspartyl-glutamate (tNAA). The ratio of SNR values between the 300 mT/m and 80 mT/m gradient systems was calculated using previously-published metabolite *T*_2_ values (Ke et al., 2002; Ganji et al., 2012):


(1) $$\begin{array}{l} S\left(\text{TE}\right)\sim S_{0}e^{-\text{TE}/T_{2,\text{metabolite}}} \end{array}$$

(2) $$\begin{array}{l} \frac{{\text{SNR}}_{300}}{{\text{SNR}}_{80}}\left(b\right)=\frac{S\left({\text{TE}}_{min,300}\left(b\right)\right)}{S\left({\text{TE}}_{min,80}\left(b\right)\right)}=\exp\frac{-{\text{TE}}_{min,300}\left(b\right)+{\text{TE}}_{min,80}\left(b\right)}{T_{2,\text{metabolite}}} \end{array}$$

where, $\frac{{\text{SNR}}_{300}}{{\text{SNR}}_{80}}\left(b\right)$ is the ratio of TE-dependent SNR values corresponding to the respective gradient systems, *S* is the signal amplitude, *S*_0_ is the signal amplitude before *T*_2_-weighting, and *T*_2, metabolite_ is the metabolite-specific *T*_2_. Note that the SNR ratio is independent of *S*_0_. The SNR ratio was calculated for a range of maximum *b*-values (and hence, TEs) for each metabolite. Apart from the pure *T*_2_ relaxation-driven reduction in SNR at longer TE, J-evolution contributes to metabolite dephasing and increases fitting uncertainties as well (Landheer et al., 2020); however, these effects were not included in our simple model, as we focused on the major singlet resonances.

### 2.2 Data acquisition

In order to evaluate measurement procedures and data processing strategies pilot data were acquired in small test group of 3 healthy participants—1 female; age 33 ± 10 years (mean ± standard deviation). Spectroscopic and structural data were collected on a research-only 3T Connectom MRI scanner, a modified 3T MAGNETOM Skyra system equipped with 300 mT/m gradients (Siemens Healthcare, Erlangen, Germany) and a 32-channel receive array head coil (Nova Medical, Wilmington, United States). The study was performed with ethics approval from the Cardiff University School of Psychology ethics review board and written informed consent was obtained from all participants. Additional Supplementary material were acquired on an isotropic diffusion phantom (NIST) (Palacios et al., 2017).

#### 2.2.1 Structural MRI

Each *in vivo* MR protocol included a Magnetization Prepared Rapid Acquisition Gradient Echoes (MPRAGE) (Mugler III and Brookeman, 1990) scan. Scanning parameters for the MPRAGE sequence were: 1mm isotropic resolution, TR = 2.3s, TE = 2ms, TI = 837ms, Flip-angle = 9°, field-of-view=256 × 256.

#### 2.2.2 DW-MRS

To reduce the impact of pulsation artefacts, all *in vivo* DW-MRS scans were cardiac triggered to avoid systolic pulsation using a pulse oximeter placed on the participant's forefinger. DW-MRS voxels were positioned using the *T*_1_-weighted MPRAGE.

In order to avoid cross-term effects from gradient overlap, diffusion gradients were applied along the physical single-gradient axes. To facilitate this, no rotations were applied to the voxels and the dimensions were adjusted to maximize the coverage of the relevant tissue volume in each case.

##### 2.2.2.1 300 mT/m vs. 80 mT/m maximum gradient strength

The DW-PRESS sequence's functionality was extended to allow precise specification of the gradients applied. This allowed us to simulate different gradient configurations on the Connectom scanner by selecting appropriate minimum rise times and the maximum gradient amplitudes, *G*_max_. While this single-scanner experimental design limited the conclusions we could draw about the performance of the Connectom compared to a different system with lower maximum gradient strength, our approach allowed comparisons to be drawn between different gradient strengths without the confounding impact of certain experimental influences. Specifically, this approach removed the risk of different voxel placement, ensured the equivalence of scanner calibrations, and allowed us to study gradient-strength differences without introducing differences in gradient non-uniformities. Here, we considered conservative limits for the gradient characteristics [slightly below the hardware and physiological limits (Setsompop et al., 2013)] for the Connectom system, which can be potentially extended and thus the maximum-achievable *b*-values increased.

For the DW-MRS protocol, we selected TEs of 116ms and 74ms for *G*_*max*_ = 80 mT/m (Prisma-like setting) and *G*_*max*_ = 300 mT/m, respectively. Diffusion weighting was applied along a single gradient axis. The diffusion-gradient configurations provided as {δ, ϵ, *a*, Δ [ms]} and shown in Figure 1A were {20.7, 0.5, 9.2, 56.2} and {7.1, 3.6, 9.2, 35.2} for 80 mT/m and 300 mT/m, respectively, giving a maximum *b*-value of ≈30000s/mm^2^. Further specification of the sequence parameters are listed in Table 1. TEs and flat-top gradient durations were rounded to the nearest *ms* by the sequence implementation, resulting in unavoidable differences in *b*-values between the two conditions. However, these deviations were minimized to the greatest extent possible, and less than 1.5% in all cases. For each gradient setting, 24 transients were acquired with water suppression, along with 8 water-unsuppressed transients. A single 22 × 20 × 22 mm^3^ DW-MRS voxel was acquired for both gradient conditions in the grey-matter rich occipital lobe (OCC) of one participant in a single scan session.

**Table 1:** Sequence parameters for the 300 and 80 mT/m gradient conditions.

	***g***_**max**_ **=300 mT/m**		***g***_**max**_ **=80 mT/m**	
	***g* [mT/m]**	***b* [s/mm^2^]**	***g* [mT/m]**	***b* [s/mm^2^]**
Gradient amplitudes and corresponding *b*-values	59	1, 257	16	1, 245
	89	2, 838	24	2, 801
	118	4, 989	32	4, 980
	177	11, 226	48	11, 204
	236	19, 957	64	19, 918
	295	31, 183	80	31, 122
TE [ms]	74		116	
δ [ms]	7.1		21.4	
Rise time [ms·m/T]	12		6 (5)	

Nominal *b*-values were calculated for the echo-times 74 ms and 116 ms for *g*_max_=300 mT/m and *g*_max_=80 mT/m, respectively.

##### 2.2.2.2 Gray matter vs. white matter

To compare tissue-type specific differences, DW-MRS voxels were acquired in grey matter (GM) and white matter (WM) rich brain regions of the other two participants with a modified protocol. In one participant, a 24 × 20 × 25 mm^3^ voxel was placed in the OCC, centred on the mid-line and as posterior as possible without including the sagittal sinus or skull, maximising the GM fraction. In a second participant, a 27 × 16 × 16 mm^3^ voxel was placed in the sub-cortical white matter of the corona radiata (CR) in the left hemisphere, maximising the WM fraction. Contrary to the previous section, three diffusion weightings were applied along each of the physical gradient axes with a single maximum gradient amplitude (295 mT/m), and reduced TE of 70 ms giving a gradient configuration of {7.1, 3.6, 9.2, 31.2} and a reduced maximum *b*-value of 21763 s/mm^2^. The 8 nominal *b*-values were 0, 557.8, 1266.2, 2260.6, 5107.4, 9099.3, 14237, and 21763 s/mm^2^ along each of the three orthogonal single-gradient axes. Both DW-MRS acquisitions otherwise used the same parameters: *TR*_*min*_ = 2, 500 ms, spectral width = 4, 000*Hz*, 24 water-suppressed, and 8 water-unsuppressed transients.

##### 2.2.2.3 Table vibration phantom scans

In order to investigate table vibrations induced by the gradient system, we acquired non-water-suppressed DW-MRS spectra in an isotropic diffusion phantom with a volume fraction of 50% polyvinylpyrrolidone (PVP, NIST). Spectra were acquired for the 300 mT/m and 80 mT/m gradient settings, with three diffusion directions, along each of the three orthogonal single-gradient physical axes. For each *b*-value, 4 transients were acquired, as well as 4 corresponding transients with inverted gradient polarity i.e., *g* = [0, ±59, ±89, ±118, ±177, ±236, ±290] mT/m. The inverted scans facilitated eddy current correction for the non-water-suppressed data, as described in the following section.

### 2.3 Data processing

#### 2.3.1 MRS data processing

A DW-MRS pre-processing pipeline—conforming to MRS consensus recommendations (Near et al., 2021)—was implemented using the Matlab-based MRS toolkit, FID-A (Simpson et al., 2017)^1^. Relative coil phasing was applied using the water-unsuppressed *b* = 0 acquisition, weighting individual coil elements based on signal-to-noise ratio (Hall et al., 2014). Motion-corrupted transients were identified using a likeliness metric, comparing FIDs to the first acquisition for each respective diffusion condition (Simpson et al., 2017). Transients which varied by more than 2 standard deviations were omitted prior to averaging to reduce the impact of motion on the final results. To minimize signal losses due to phase and frequency drift, spectral registration (Near et al., 2015) was used to align individual transients for each diffusion condition, separately. The resulting spectra were then manually inspected for residual water/lipid or motion contamination, automated data quality cutoffs were used for tNAA full-width at half-maximum greater than 0.1 PPM and metabolite SNR less than 3 (Wilson et al., 2019).

Tarquin (Wilson et al., 2011) V4.3.10 was used for linear-combination modeling (LCM), with TE-specific simulated basis sets including -CrCH2 (relaxation correction basis function), alanine (Ala), aspartate (Asp), creatine (Cr), GABA, glycerophosphocholine (GPC), glucose (Glc), glutamine (Gln), glutathione (GSH), Glu, glycine (Gly), mI, lactate (Lac), NAA, N-acetylaspartylglutamate (NAAG), phosphocholine (PCh), phosphocreatine (PCr), phosphorylethanolamine (PE), scyllo-inositol (sI), and taurine (Tau). Reported metabolites are total creatine (tCr = Cr + PCr), total NAA (tNAA = NAA + NAAG), and total choline (tCho = Cho + GPC). Additional basis functions were incorporated for macromolecular and lipid resonances, with the baseline approximated by a Gaussian window function. The extracted metabolite amplitudes and Cramér-Rao lower bounds (CRLBs) were then used for diffusion modeling. Water phantom scans were processed without LCM, with the water signal amplitude quantified by taking the magnitude of the first point of the complex FID.

#### 2.3.2 Eddy current correction

While the DW-PRESS sequence was designed to minimize eddy current artefacts using a bipolar gradient scheme (Branzoli, 2015), further eddy-current correction was required, particularly for the highest *b*-values. For *in vivo* data, we performed eddy current correction using a non-water-suppressed reference scan. The signal of the water-suppressed scan, *S*_met_(*t*) can be divided by the signal of the non-water-suppressed reference *S*_ref_(*t*) to remove the eddy-current-related phase, ϕ_eddy_, (Klose, 1990):


(3) $$\begin{array}{l} S_{\text{met}}\left(t\right)=|S_{\text{met}}|e^{i\left(\phi_{\text{met}}\left(t\right)+\phi_{\text{eddy}}\left(t\right)\right)} \end{array}$$

(4) $$\begin{array}{l} S_{\text{ref}}\left(t\right)=|S_{\text{ref}}|e^{i\phi_{\text{eddy}}\left(t\right)} \end{array}$$

where the phase information of the MR spectrum, ϕ_met_, is retained. To mitigate issues resulting from the attenuation of the water signal at higher *b*-values, the relevant phase correction term, ϕ_eddy_ was extracted from the reference scan using an LPSVD (Vanhamme et al., 1998), improving robustness to the noisier high-*b*-value water transients.

The Klose et al. method was used for all *in vivo* data in our study. Testing revealed satisfactory performance, even at high *b*-value; however, this might not be the case for all types of acquisition. To supplement this approach, we also demonstrate a second method of *post-hoc* eddy current correction in a nickel-doped water phantom, using gradient polarity inversion (Lin et al., 1994), whereby alternating transients of a particular diffusion gradient strength are acquired with opposite gradient polarity. This reverses the phase development due to eddy currents, such that:


(5) $$\begin{array}{l} S_{\text{invert}}\left(t\right)=|S\left(t\right)|e^{i\phi \left(t\right)-i\phi_{\text{eddy}\left(t\right)}} \end{array}$$

(6) $$\begin{array}{l} S\left(t\right)S_{\text{invert}}\left(t\right)=|S\left(t\right){|}^{2}e^{i\phi \left(t\right)} \end{array}$$

where, ϕ is the phase, ϕ_eddy_ is the phase specifically resulting from eddy currents, and *S* and *S*_invert_ are the signals resulting from the initial and inverted gradient polarities, respectively. Multiplying the resulting time-domain signals removes phase evolution due to eddy currents.

#### 2.3.3 Gradient non-uniformity correction

A mask representing the DW-MRS voxel was created in the anatomical image space using SPM12 (Penny et al., 2011; Edden et al., 2014). A vendor-supplied spherical harmonic description of the spatial dependence of the field generated by each gradient coil was used to assess geometric deviations of the voxel. A coil tensor, ***L***, was computed by taking the partial derivatives of the field—normalized by the nominal gradient strength—where the elements of the coil tensor contain the spatially varying deviations for each of the gradient axes. The coil tensor was subsequently used to compute the effective *b*-matrix and *b*-value from the nominal ones, i.e., $B_{eff}={LBL}^{T}$ and *b*_*eff*_ = trace(***B***_*eff*_) (Bammer et al., 2003). Nominal *b*-values were estimated using in-house software written in Matlab (Mathworks, Natick, MA, USA), then subsequently, the nominal *b*-values were corrected for gradient non-uniformities (Bammer et al., 2003). This gave rise to a distribution of corrected *b*-values for each diffusion condition—rather than the single nominal value. The corrected mask was used in conjunction with the corrected *b*-values, to ascertain the effective *b*-value within the DW-MRS voxel.

#### 2.3.4 Diffusion measures

Data were fitted in the low (*b* < 3, 000*smm*^−2^) and intermediate (*b* < 9, 500*smm*^−2^) *b*-value range with a mono-exponential and kurtosis diffusion representation, respectively, and over the full *b*-value range with an astrocylinder model (fully dispersed cylinders). All diffusion fitting was performed in Matlab 2021b using trust-region reflective optimisation. The inverse of the metabolite CRLBs was used to weight each data point, to reduce the impact of individual poorly-fit spectra on quantification.

##### 2.3.4.1 Diffusion representation

Metabolite ADCs were estimated for each direction independently. The reported metabolite amplitudes for each *b*-value were modeled using two approaches. Firstly, all *b*-values below 3, 000 s/mm^2^ were fit using a mono-exponential decay:


(7) $$\begin{array}{l} \ln\left(S\right)=\ln\left(S_{0}\right)-b\cdot ADC_{e} \end{array}$$

where ln(*S*) is the natural logarithm of the fitted metabolite amplitude, and *b* is the *b*-value. *S*_0_ and *ADC*_*e*_ are the non-diffusion-weighted signal amplitude and apparent diffusion coefficient (where _*e*_ indicates the *ADC* from a mono-exponential fit), respectively. Non-mono-exponential behavior was observed beyond this *b*-value range, so *b*-values up to 9, 500 s/mm^2^ were fit using the diffusion kurtosis representation (Jensen et al., 2005; Yablonskiy and Sukstanskii, 2010):


(8) $$\begin{array}{l} \ln\left(S\right)=\ln\left(S_{0}\right)-b\cdot ADC_{k}+\frac{1}{6}{\left(b\cdot ADC_{k}\right)}^{2}\cdot K \end{array}$$

where *ADC*_*k*_ is the apparent diffusion coefficient from the kurtosis representation and *k* is the kurtosis. To stabilize fitting, *ADC*_*e*_ estimates from the mono-exponential representation were used to initiate the fit of the kurtosis representation.

Following gradient non-uniformity correction, each single nominal *b*-value, *b*_*i*_, is replaced with a distribution of corrected *b*-values (denoted *b*_*i*,_eff__*j*__). To investigate the impact this had on metabolite diffusion measures, we estimated mono-exponential fits considering the distribution of *b*-values within the DW-MRS voxel:


(9) $$\begin{array}{l} \left[\begin{array}{c} \ln S(b_{1})\\ \vdots \\ \ln S(b_{1})\\ \vdots \\ \ln S(b_{N}) \end{array}\right]=\left[\begin{array}{cc} 1 & -b_{1,{\text{eff}}_{1}}\\ \vdots & \\ 1 & -b_{1,{\text{eff}}_{M}}\\ \vdots & \\ 1 & -b_{N,{\text{eff}}_{M}} \end{array}\right]\cdot \left[\begin{array}{c} \ln S_{0}\\ ADC_{\text{d}} \end{array}\right] \end{array}$$

where *ADC*_d_ is the apparent diffusion coefficient (_*d*_ indicates that it is estimated from a distribution of *b*-values), and the signal of the *i*^*th*^ *b*-value, *S*(*b*_*i*_), is repeated across *M* voxels at 1 mm^3^ isotropic resolution (i.e., the resolution of the MPRAGE).

##### 2.3.4.2 Diffusion modeling

Code adapted from the Multidimensional diffusion MRI (MD-dMRI) analysis framework was used to implement a single-compartment model of fully-dispersed cylinders (astrocylinders) for bipolar diffusion encoding, in agreement with DW-MRS recommendation (Nilsson et al., 2017, 2018; Ligneul et al., 2023). To rate model performance and investigate ultra-high *b*-values with Connectom settings (higher SNR, shorter TE, and diffusion-time) the corrected Akaike information criterion (AICc) was used. The model parameters include the signal amplitude *S*_0_ at *b* = 0, the free diffusivity *D*_0_ and the cylinder radius *R*_*C*_ with boundaries 0 ≤ *R*_*C*_ ≤ 20μ*m*. Moreover, fitting included outlier detection to remove data-points potentially affected by motion by iteratively removing a single *b*-value from the fitting and identify the fit with the lowest root-mean-square-error (c.f., Supplementary Figures S3, S4). In the case of multi-directional data, the directional average of the signal was used for fitting to mitigate effects from tissue anisotropy. The fitting uncertainties of the parameters were estimated by residual bootstrapping with 250 random noise realizations (Jelescu et al., 2022).

## 3 Results

### 3.1 SNR simulations

Figures 1B, C shows the results of the theoretical investigation into the relationship between maximum-achievable *b*-value and minimum-achievable TE for the DW-PRESS sequence, as well as the related SNR simulation results. As anticipated, ultra-strong gradients allow for a far shorter TE for a given *b*-value, a benefit which becomes even more apparent as the required maximum *b*-value increases. Moreover, one can see from the color bar that the minimum achievable diffusion time for a given maximum *b*-value is strongly related to the maximum gradient strength. In terms of SNR, we found that tCr—with the shortest *T*_2_ of the metabolites considered—had the largest SNR gains. For a maximum *b*-value of 31 000 s/mm^2^, our simulations suggest a potential SNR improvement of 50%. Likewise, for tNAA and tCho, our simulations suggest an expected SNR gain of about 10% and 21% moving from 80 mT/m to 300 mT/m, respectively.

### 3.2 Eddy currents

**Figure 2:**
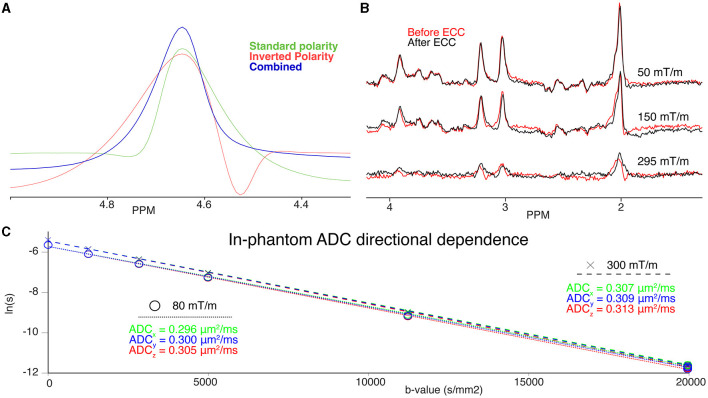
**(A)** Phantom water spectra acquired with a given gradient polarity (green), inverted gradient polarity (red), and the combined, eddy-current corrected spectrum (blue) **(B)** Three examples of spectra before (red) and after Klose eddy-current correction (black) at 50 mT/m (top), 150 mT/m (middle), and 295 mT/m (bottom). **(C)** DW-MRS quantification of the isotropic diffusion phantom. Individual diffusion directions (x, green; y, blue;, z, red) are plotted on top of each other for the 300 mT/m (dashed fit line above, “x”) and 80 mT/m (dotted fit line below, “o”) acquisitions. The individual quantified ADCs are also noted in the colour corresponding to the direction acquired. Note the strong overlap between directions, and vertical offset between gradient conditions.

Figure 2 shows a summary of sequence validation results, including examples of gradient-polarity-inversion eddy current correction (ECC) in a phantom (Figure 2A), and examples of water-reference-based ECC *in vivo* (Figure 2B). Although bipolar diffusion gradients were applied in the water PVP phantom, Figure 2A shows clear eddy current artifacts for both positive and negative gradient polarity configurations. However, by combining both datasets, effects from eddy currents can be largely prevented. Apart from the benefits of limiting eddy-current artifacts, combining consecutive transients with inverted gradient polarity also reduces contributions from linear gradient cross-terms. This allows for an accurate estimation of the *b*-value, even without taking slice-selection, and crushing gradients into account. If no gradient polarity inversion is used, one has to calculate the real *b*-value directly from the full gradient chronograms (Mattiello et al., 1997). For the *in vivo* acquisitions, where only a single gradient polarity configuration was used, the effects from eddy currents are clearly visible from Figure 2B (red line, Before ECC). This effect is more pronounced at higher *b*-values, where steeper gradients give rise to stronger induction currents. However, when the acquired water reference is used for ECC (black line, After ECC) non-linear phase distortions from eddy-currents can be widely prevented. Despite large differences in diffusion gradient characteristics, MRS fit residuals were comparable between the 80 mT/m and 300 mT/m acquisitions (Supplementary Figure S1). The mean ratio between the fit residual and the noise level—as reported by Tarquin—was 2.98 and 3.04 for the 300 and 80 mT/m acquisitions, respectively. The comparability of the residuals alludes to a lack of significant differences in modeling performance, despite the larger eddy current artifacts induced when ultra-strong gradients are applied.

### 3.3 Gradient non-uniformities

**Figure 3:**
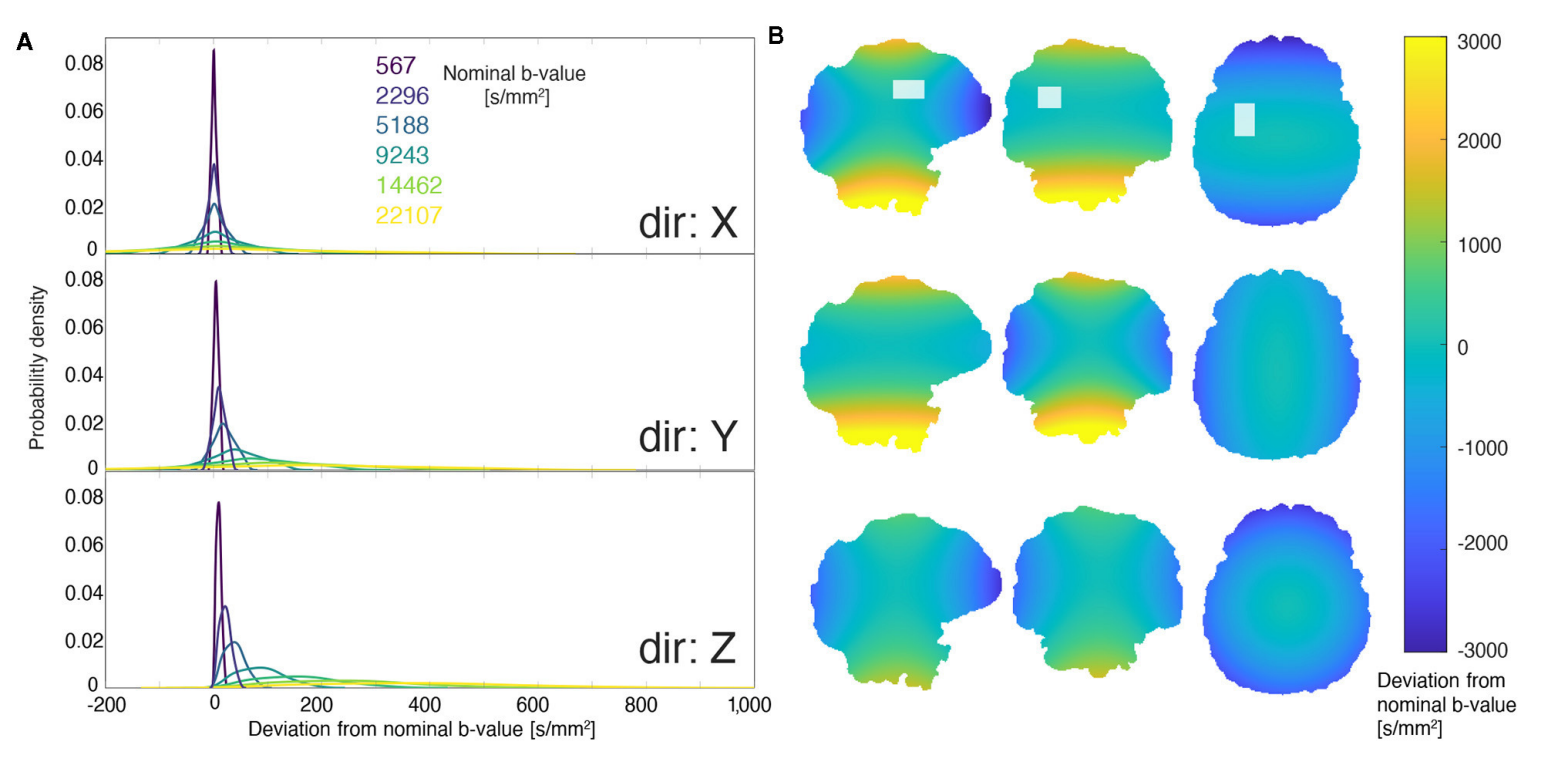
Deviation from nominally specified *b*-values due to gradient non-uniformity. **(A)** The distribution of deviation from the nominal *b*-value within the voxel. From dark blue to yellow, the distributions correspond to nominal *b*-values of 567, 2,296, 5,188, 9,243, 14,462, and 22,107 s/mm^2^. Higher *b*-values exhibit broader distributions of deviations. **(B)** An example of the spatial distribution of deviations from a nominal *b*-value of 22,107 s/mm^2^. The rows correspond to different gradient directions (consistent with the first panel), and the columns represent orthogonal slices. The images in the top row are overlaid with the DW-MRS voxel location used to determine the distributions shown in the left panel.

Deviations in the voxel mask geometry and *b*-values due to gradient non-uniformities were corrected by taking the nonlinear spatial gradient profiles into account. While voxel deformations made little-to-no difference to the voxel volume and position (< 1% deviation), *b*-values were affected significantly. This is perhaps expected, as unlike spatial encoding, *b*-values are driven by the squared gradient amplitude. Figure 3 shows the magnitude and spatial distribution of deviations from the nominally-specified *b*-value due to gradient non-uniformities. While the fractional deviation from the nominal *b*-value is constant, the actual deviation increased with gradient strength. A relatively narrow distribution is observed about zero at the lowest non-zero *b*-value and a much wider, non-zero-centered distribution is seen for the highest *b*-value. For the smallest nominal *b*-value of 567 s/mm^2^, the mean (standard deviation) of the distributions of corrected *b*-values in *x*, *y*, and *z* were 563 (5), 567 (5), and 559 (5) s/mm^2^, respectively, and for the largest nominal *b*-value of 22,107 s/mm^2^–21,948 (198), 22,137 (185), 21,802 (199) s/mm^2^, respectively. The mean deviation—calculated by taking subtracting nominal *b*-value from the corrected one—were −4, 0, and −8 s/mm^2^ for 557.8 s/mm^2^, and –159, 30, and –305 s/mm^2^ for a nominal value of 21,763 s/mm^2^.

**Figure 4:**
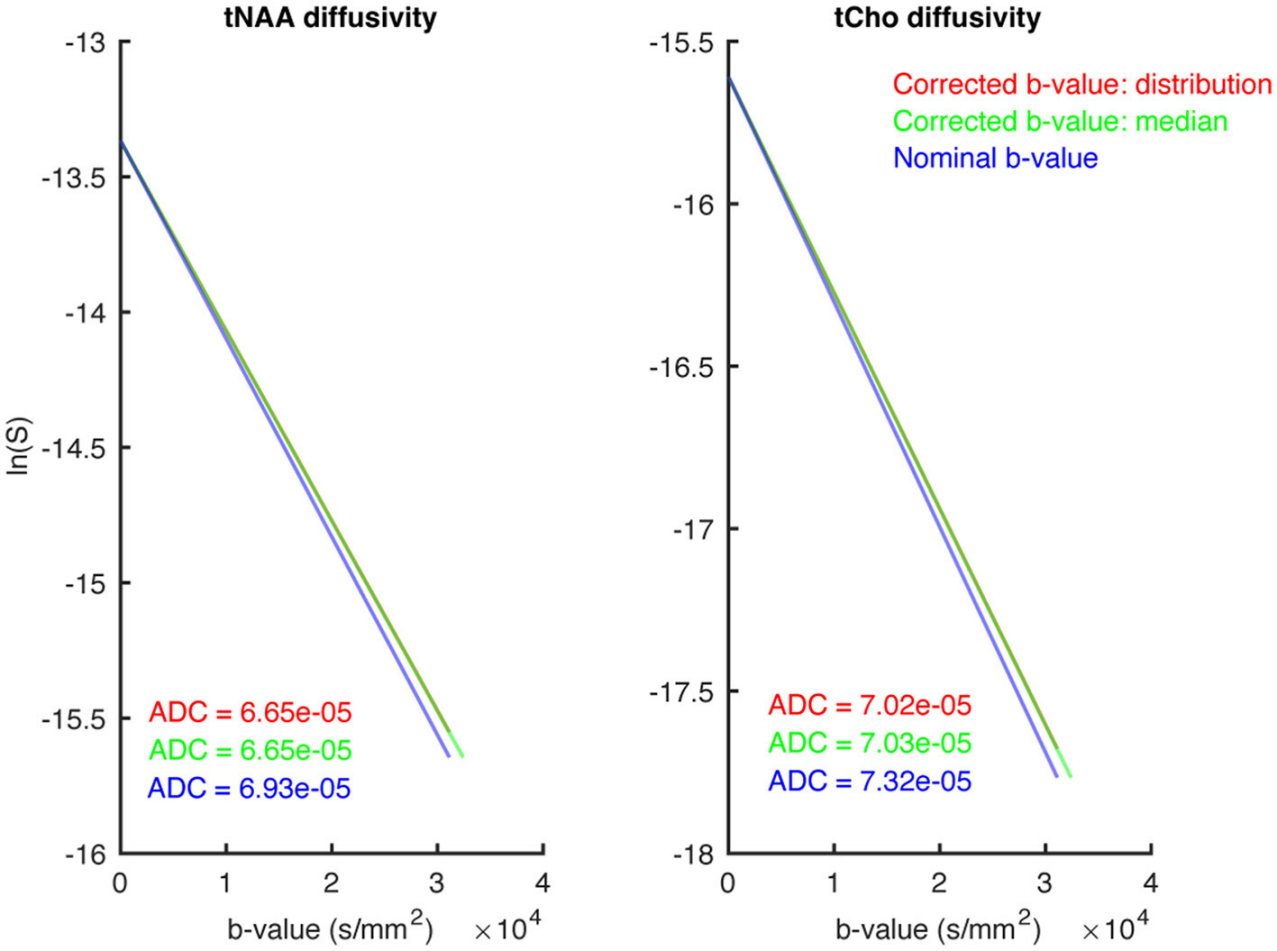
This figure shows three different approaches to representing the *b*-value during mono-exponential modelling. Directly fitting using the nominally-specified *b*-value (blue), fitting using the median of the distribution of gradient-non-linearity-corrected *b*-values (green), and fitting using the full distribution of corrected *b*-values (red). The resulting ADCs are shown in their respective colours. The **(left)** shows the tNAA diffusivity, while the **(right)** shows tCho.

Figure 4 shows *in vivo* mono-exponential fitting results if *b*-values were not corrected for non-uniformity (blue); corrected for non-uniformity, fitting the the full in-voxel distribution (red); and corrected for non-uniformity using the median of the distribution for fitting (green). After correction, we observed no significant difference in the estimated ADCs when either using the full *b*-value distribution or the median of that distribution, but overestimated ADCs in the absense of non-uniformity correction (approximately 4% faster diffusion). Thus, in the subsequent analysis, the median *b*-value was used to inform ADC, kurtosis, and microstructural fitting.

### 3.4 Comparison of 80 and 300 mT/m acquisitions

**Figure 5:**
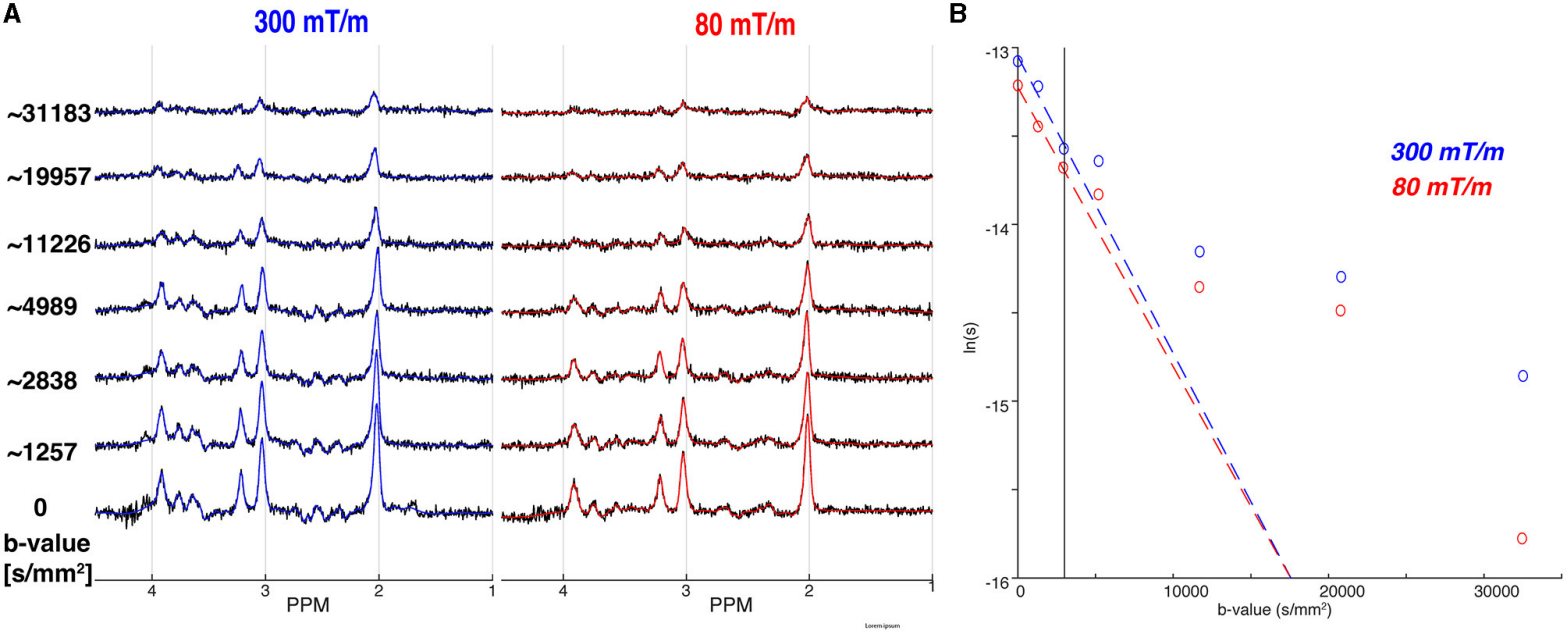
Comparison of the 300 mT/m acquisition to the 80 mT/m acquisition. **(A)** The individual spectra (black) for all *b*-values are overlaid by the corresponding MRS fits for the 300 mT/m (blue) and 80 mT/m (red), respectively. The approximate *b*-values are noted on the left, with full details in Table 1. **(B)** The mono-exponential (dashed coloured line) fits for the 300 mT/m (blue) and 80 mT/m acquisition (red) for tNAA. The vertical line indicates the *b*-value data exclusion threshold for mono-exponential modeling.

Figure 5A shows the individual *in vivo* spectra and LCM fitting results left with the Connectom (*G*_*max*_ = 300*mT*/*m*) and right with the Prisma (*G*_*max*_ = 80*mT*/*m*) settings. Overall spectral SNR of all acquisitions was well above minimum consensus recommendations, even for the highest *b*-value. The mean SNR gain for tNAA—calculated by taking the ratio of the metabolite peak amplitude to the standard deviation of the noise—was 1.16, with a 16% increase in SNR when ultra-strong gradients are used. Likewise, for the other major metabolites, we found an SNR gain of 1.36 for tCr, and 1.27 for tCho. To further elucidate the results, we expanded our SNR calculations to examine J-coupled metabolites—Glu and mI (Wyss et al., 2018)—and found similar SNR gains: a predicted 30% improvement for Glu was experimentally verified, and a 29% improvement was observed for mI, compared to the expected 31%.

The mean full-width half-maximum of tNAA was found to be 6.2 Hz across both acquisitions, indicating a good shimming and linewidth for LCM quantification. The tNAA CRLBs were all less than 27%—even including the highest *b*-values—with a mean value of 17% across all *b*-values. Similarly, the mean CRLB for tCr was 14%, with all-but-one value falling below 22%. CRLBs were higher for tCho with 4 acquisitions greater than 27%. Only a single transient was rejected by the motion corruption metric—for the 300 mT/m acquisition at the highest *b*-value. Full data quality measures are reported in Supplementary Table S2.

Figure 5B shows the tNAA diffusion decay fitted to a mono-exponential for the single diffusion direction acquired in the OCC. For the mono-exponential representation, the apparent diffusion coefficients of tNAA were ADC_e, 300_ = 0.168 μm^2^/ms, and ADC_e, 80_ = 0.158 μm^2^/ms for 300 and 80 mT/m, respectively. Results for the other metabolites—tCho and tCr—are reported in Table 2. The Kurtosis representation was not included for these data, as only a single data point fell in the intermediate *b*-value range of 3, 000–9, 500 s/mm^2^.

**Table 2:** Listing of the *in vivo* results applying a mono-exponential and kurtosis representation for the three major metabolites.

**Experiment**	**Metabolite**	**ADC_*e*_ [μm^2^/ms]**	**adj.${\text{R}}_{e}^{2}$**	**ADC_*k*_ [μm^2^/ms]**	**K**	**adj.${\text{R}}_{k}^{2}$**
300mT/m	tNAA_Z_	0.168	0.94	-	-	-
	tCho_Z_	0.116	0.93	-	-	-
	tCr_Z_	0.125	0.73	-	-	-
80mT/m	tNAA_Z_	0.158	0.99	-	-	-
	tCho_Z_	0.184	0.69	-	-	-
	tCr_Z_	0.184	0.75	-	-	-
CR voxel	tNAA_X_	0.146	0.99	0.142	2.226	0.98
	tNAA_Y_	0.122	0.99	0.170	1.855	0.98
	tNAA_Z_	0.153	0.97	0.166	1.622	1.00
	tCho_X_	0.223	1.00	0.204	1.466	0.96
	tCho_Y_	0.185	0.81	0.211	1.758	0.97
	tCho_Z_	0.112	0.45	0.101	0.192	0.94
	tCr_X_	0.191	0.97	0.231	1.589	0.99
	tCr_Y_	0.158	0.89	0.167	1.383	0.99
	tCr_Z_	0.177	0.89	0.136	1.014	0.96
OCC voxel	tNAA_X_	0.128	0.93	0.180	1.569	0.98
	tNAA_Y_	0.153	0.42	0.194	2.147	0.82
	tNAA_Z_	0.096	0.79	0.122	1.542	0.98
	tCho_X_	0.079	0.47	0.104	1.913	0.94
	tCho_Y_	0.211	0.77	0.154	1.557	0.84
	tCho_Z_	0.209	0.92	0.160	1.518	0.93
	tCr_X_	0.181	0.78	0.204	1.477	0.96
	tCr_Y_	0.202	0.36	0.231	1.628	0.80
	tCr_Z_	0.156	0.89	0.173	1.070	0.99

The Experiment column delineates the acquisition and the metabolite column specifies the metabolite considered, with the subscript indicating the diffusion direction. The obtained-R^2^ for each model is included to evaluate the overall fitting quality of the diffusion decay.

Table 3 lists, in the upper rows, the estimated microstructural properties derived from the astrocylinder model comparing the 300 and 80 mT/m settings acquired in the OCC. The estimated AICc's are similar between 80 and 300 mT/m settings. It is interesting to note that D_0_ is, by average, higher for the Prisma settings, which is also apparent from the signal attenuation at low *b*-values in Supplementary Figure S3, and in line with the estimated ADCs. Moreover, R_C_ tends toward zero for tNAA for the Prisma configuration.

**Table 3:** Listing of the *in vivo* results of microstructural measures (free diffusivity D_0_, cylinder radius R_C_) estimated from an astrocylinder model.

**Experiment**	**Metabolite**	**D_0_ [*μm*^2^*ms*^−1^]**	**R_C_ [*μm*]**	**AICc**
300 mT/m	tNAA_Z_	0.33 ± 0.09	2.2 ± 0.1	−51.1
	tCho_Z_	0.39 ± 0.06	1.4 ± 0.3	−59.4
	tCr_Z_	0.41 ± 0.07	1.6 ± 0.4	−57.8
80 mT/m	tNAA_Z_	0.52 ± 0.08	0.0 ± 0.0	−59.0
	tCho_Z_	0.92 ± 0.14	2.4 ± 0.9	−56.4
	tCr_Z_	0.72 ± 0.16	2.7 ± 1.0	−55.3
CR voxel	tNAA_avg_	0.43 ± 0.07	2.2 ± 0.4	−73.1
	tCho_avg_	0.48 ± 0.06	1.6 ± 0.7	−76.1
	tCr_avg_	0.46 ± 0.05	2.6 ± 0.2	−82.1
OCC voxel	tNAA_avg_	0.39 ± 0.05	1.5 ± 0.3	−76.5
	tCho_avg_	0.36 ± 0.04	0.0 ± 0.7	−72.3
	tCr_avg_	0.57 ± 0.07	1.9 ± 0.2	−74.6

The double-lined-column delineation shows, in the upper half, the comparison of the 300 and 80 mT/m settings where diffusion-encoding was applied only along the z-direction, and in the lower half, the comparison of the averaged diffusion metrics over three orthogonal diffusion-directions in the white matter rich corona radiata (CR) and grey matter rich occipital lobe (OCC) using the 300 mT/m setting. The fitting results can be found in Supplementary Figure S3.

### 3.5 Grey matter and white matter

**Figure 6:**
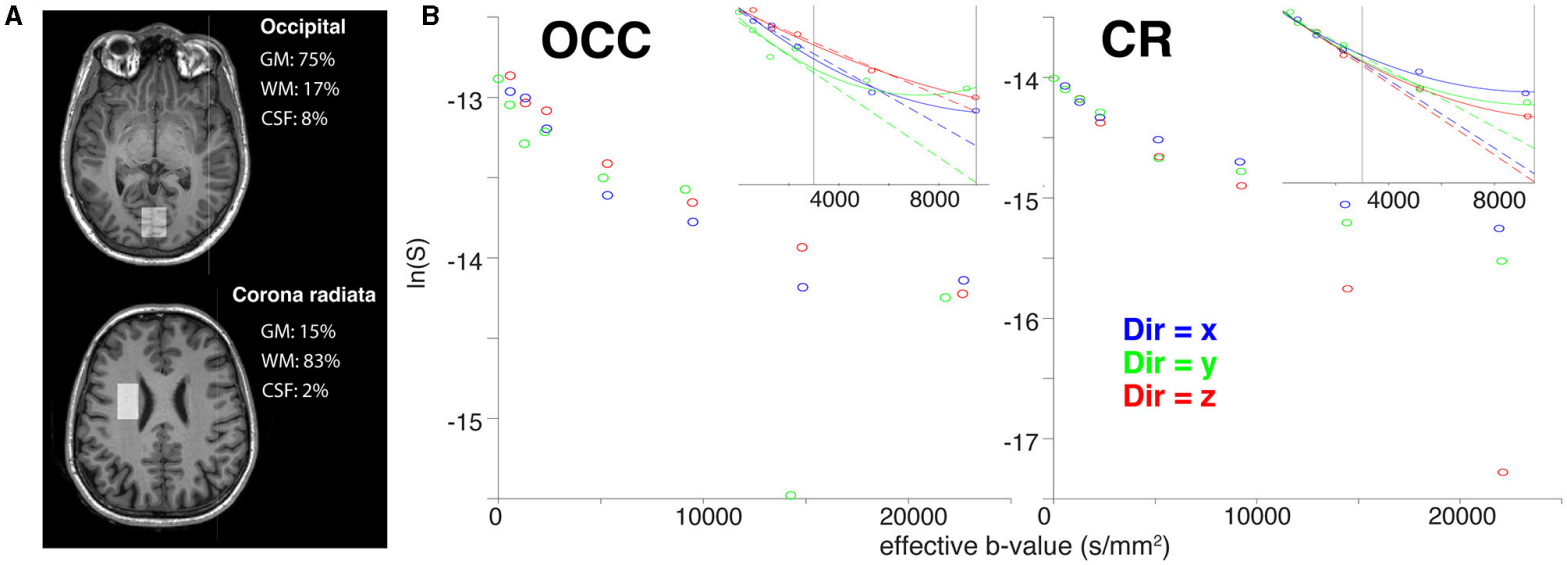
Results of the *in vivo* grey and white matter acquisitions. **(A)** *T*_1_-weighted images are overlaid with tissue-specific segmentation masks for grey matter (GM), white matter (WM), and CSF in the occipital (above) and white matter (below) voxels. Grey matter, white matter, and CSF voxel fractions are reported in the corresponding text. **(B)** The three tNAA diffusion decays for the OCC and CR voxels. Mono-exponential (dashed lines) and kurtosis (solid lines) fits are shown for the x (blue), y (green), and z (red) directions. The vertical lines indicate the *b*-value thresholds of data exclusion for mono-exponential and kurtosis modelling, respectively.

Figure 6A shows the voxel positions and tissue segmentation in the OCC and CR estimated from 3D T_1_ MPRAGE (Penny et al., 2011; Edden et al., 2014). The OCC voxel contained predominantly grey matter (GM/WM/CSF: 75/17/8 %) and the CR voxel contained predominantly white matter (GM/WM/CSF: 15/83/2 %). Spectral quality was generally high for both voxels. The minimum SNR—as measured by the maximum point of tNAA—was 10.9 in the OCC and 8.9 in the CR at *b* = 0. The mean FWHM across the DW-MRS acquisitions was 5.65 Hz for the OCC voxel, and 6.31 Hz for the CR voxel, well within consensus recommended limits (Wilson et al., 2019). The tNAA CRLBs ranged from 3.4–9.7% for the OCC, and 5.1–51.9% for the CR, with difference between voxels likely driven by the lower SNR in the CR voxel, or perhaps reflects increased variability due to the proximity of the CR voxel to the ventricles, as previously reported (Genovese et al., 2021a). We also noted lower data quality for the OCC voxel in the y-direction, which exhibited lower SNR and increased CRLBs, compared to the other two directions. Regarding motion-corruption, we excluded a greater number of transients in the data sets for the GM/WM comparison than in the previous analysis. In all cases, no more than 2 transients were removed per diffusion weighting, with one exception for the CR voxel, where 3 transients were removed for a single diffusion condition. All spectra and fits are shown in Supplementary Figure S2. Figure 6B shows the fitting results for tNAA in the OCC and CR voxels. Full data quality measures are reported in Supplementary Table S2.

Metabolite ADC_*e*_ for the mono-exponential representation and ADC_*K*_ and K for the kurtosis representation are reported in Table 2 in the lower rows for each individual diffusion-encoding direction. The mean directional-averaged ADC_e_s for tNAA/tCho/tCr are 0.14/0.17/0.18μ*m*^2^*ms*^−1^ in the CR and 0.13/0.17/0.18μ*m*^2^*ms*^−1^ in the OCC. For ADC_K_ slightly higher values were found with 0.16/0.17/0.18μ*m*^2^*ms*^−1^ in the CR and 0.17/0.14/0.20μ*m*^2^*ms*^−1^ in the OCC, and corresponding Kurtosis values of 1.90/1.14/1.33 in the CR and 1.75/1.66/1.39 in the OCC.

The estimated microstructural properties for the astrocylinder model are listed in Table 3 in the lower rows for the voxels placed in the WM-rich CR and GM-rich OCC acquired with Connectom settings. The AICc's are comparable between these two brain regions and do not indicate greater model validity in either WM or GM.

### 3.6 Table vibration

Figure 2C shows the signal decay for the isotropic diffusion phantom acquired along the three orthogonal directions x, y, and z for the Connectom and Prisma settings. The ADC for both settings only shows a slightly faster diffusion along the z-direction, which may point to stronger table vibrations along z. However, the overall estimated ADCs are well in line with the calibration value of 0.293 μm^2^/ms at 22°*C*. Considering our results *in vivo* shown in Figure 6 we found a stronger signal decay for tNAA, but also tCr and tCho (not shown), in the CR when diffusion-encoding is applied along the z-direction at ultra-high b-values.

## 4 Discussion

In this study, we investigated the practical feasibility of DW-MRS on a Connectom MR scanner equipped with 300 mT/m gradients to measure metabolite diffusion at ultra-high *b*-values in the human brain. By comparing these results between clinical and ultra-high gradient amplitudes, we show the benefits of new gradient systems for DW-MRS, but also present strategies to mitigate the impact of methodological challenges.

### 4.1 SNR simulations

Simulated SNR benefits of applying ultra-strong gradients were mostly in line with experimental measurements *in vivo*. Comparing the Prisma (*G*_*max*_ = 80 mT/m) and Connectom (*G*_*max*_ = 300 mT/m) settings, the theoretical SNR gain of 10% for tNAA was even exceeded experimentally and a 16% improvement was achieved. A similar pattern is observed for tCho, where experimental SNR gains exceed predictions by 7 percentage points. J-coupled metabolites mI and Glu were approximately in-line with expectations. For tCr, while we still report an increase in the SNR at 300 mT/m, the magnitude of the SNR increase is not as large as predicted by simulation—36% vs. the expected 50%. This variation from expected SNR gains is attributed to inaccuracies in the assumed T_2_, but further verification would require acquisition of T_2_ relaxometry alongside the DW-MRS. Interestingly, two studies that investigated this—in humans and mice respectively—found that tCr was the only metabolite that showed a slight dependence of ADC on TE (**?**Mougel et al., 2022).

In general, SNR should be carefully considered in the context of diffusion imaging (Ellegood et al., 2011). The SNR dependence of MRS reconstruction and fitting methods can lead to anomalous signal decay, particularly at higher *b*-values, making it an important consideration for high-*b*-value DW-MRS. The shorter TE afforded by ultra-strong gradients improved SNR and reduced CRLBs, but strict consideration of SNR and MRS fit uncertainty is still necessary.

### 4.2 *In vivo* results

Previous work (Ronen et al., 2014) found tNAA diffusivity values of 0.076 and 0.34 μm^2^/ms for diffusion gradients orthogonal and parallel to the main fibre orientation of the corpus callosum, respectively. The DW-MRS voxels considered in this study were arranged to contain predominantly grey or white matter, but inevitably contained mixed fibre orientations. Our measured ADCs fell within the range reported by Ronen et al. (2014), with our lowest ADC reported in grey matter (0.096 μm^2^/ms) and the highest in kurtosis model of white matter (0.194 μm^2^/ms).

Furthermore, higher ADCs in GM compared to WM have been previously reported (Ellegood et al., 2011; Kan et al., 2012; Najac et al., 2016) and this finding is confirmed by our results for tNAA in our grey-white matter comparison, where we find a mean ADC of 0.126 μm^2^/ms for the OCC GM voxel, and 0.140 μm^2^/ms for the CR WM voxel. Kurtosis value ranges for tNAA (1.5–2.2), tCr (1.0–1.6), and—excluding one low-kurtosis fit (0.192)—tCho (1.5–1.9) fall within previously reported diffusion-time-dependent ranges for metabolites (Döring et al., 2023). Lower AICc values were found in the data averaged over multiple diffusion directions. This difference could potentially be attributed to a higher SNR or better agreement with the model, resulting from powder averaging. Furthermore, we examined the data to assess the feasibility of employing more extensive modeling strategies, specifically using a two-compartment model as described in Supplementary Figure S4 and Supplementary Table S1. However, the estimated AICc values were consistently were higher.

We report a 6% decrease in the tNAA ADC of 80 mT/m acquisition, compared to the 300 mT/m acquisition. Conversely, a significant increase was noted for the other two metabolites (tCho, tCr), in line with an even more pronounced increase in the free diffusivity *D*_0_. Although the goodness-of-fit was lower for the 80 mT/m configuration, a possible explanation could be that the longer TE reduces signal contributions from restricted compartments—such as organelles—and consequently increases ADCs and *D*_0_s. Previous studies within a TE range of 35 to 70 ms have not found a correlation between metabolite diffusion and T_2_ relaxation, but others at 7T and longer TE report on faster diffusion with increasing TE (**?**Ligneul et al., 2023). This is in line with our results for tCho and tCr indicating faster diffusion when TE increases from 74 to 116 ms, although T_2_s were different due to the lower field-strength and diffusion-times due to different maximum gradient amplitudes. Moreover, despite the higher SNR, shorter TE, and shorter diffusion-times achieved with the 300 mT/m settings, AICc's indicate similar model support.

It is important to note that the relatively small size of our volunteer group limits the confidence with which we can draw conclusions about our quantitative analyses. Uncertainty in the MRS modeling procedures—due to a myriad of factors, including lineshape distortion, motion, uncharacterized signals from macromolecules, and issues in baseline characterisation—will affect the measured diffusion properties necessitating a larger participant population to achieve sufficient statistical power. Here, we utilize the Cramer-Rao lower bounds, and weight the fitting accordingly in an attempt to mitigate this. However, in the future it might be beneficial to use metabolite-cycling to further reduce motion and eddy-current artifacts (Döring et al., 2018), and expanding the sample size of the study.

### 4.3 Eddy currents

Diffusion-weighted imaging and spectroscopy use strong gradients to achieve the desired diffusion weighting. Eddy currents generated during ramp up and down times can cause time-dependent frequency variations in the time domain and consequently distort the MR spectra. Correcting for eddy-current effects is vital in MRS—and particularly so for DW-MRS—in order to accurately reflect lineshape distortions while modeling MR spectra. Furthermore, as per Faraday's law, the magnitude of the induced eddy-current effects increase with the applied gradient strength, further compounding their relevance in the context of ultra-strong gradient DW-MRS. Thankfully, in the particular case of the Siemens Connectom scanner, the shield coil design minimizes the relative size of eddy current effects. Setsompop et al. (2013) reported that a 7.5-fold increase in gradient amplitude on the Connectom scanner corresponded to just a 2–3-fold eddy current increase, in absolute terms. Our sequence, acquisition scheme, and post processing pipeline further reduce the impact of eddy currents. Our DW-PRESS sequence utilized a bipolar diffusion gradient scheme, which has the potential to reduce eddy current effects on the acquired DW-MR spectra (Alexander et al., 1997; Branzoli, 2015). We further applied two methods of post-hoc eddy current correction.

Our *in vivo* DW-MRS protocols included unsuppressed water-signal, which we used to correct for eddy-current-induced phase and frequency shifts (Klose, 1990). Though this method requires acquisition of only few additional spectra, faster water diffusion may have reduced the effectiveness of this method at the highest *b*-values applied in this work. Therefore, we also demonstrate a second approach (Lin et al., 1994). For this, we inverted the polarity of diffusion-weighting gradients for half of metabolite spectra acquired at the highest *b*-values. The inversion of diffusion gradient polarities inverts the phase of the generated eddy currents, while the intrinsic signal phase and the magnitude of the diffusion-weighting are expected to remain unchanged. No additional acquisitions are required in this case. However, it should be noted that motion corruption of individual transients would necessitate the pairwise removal of the corresponding inverted diffusion condition, potentially doubling motion-related transient exclusions. Furthermore, our implementation of the method did not include the inversion of slice-selective gradients, which may explain the residual asymmetry of the lineshapes in our combined spectra.

The quality of eddy current correction of the above methods relies on the quality of the acquired water and metabolite spectra. A lot of work has been done to monitor the field perturbations in presence of diffusion gradients and their effect of the readout trajectory and the consequent deterioration in image quality in DW-MRI (Chan et al., 2014; Wilm et al., 2015). External probes could be a valuable tool in characterizing and correcting for the eddy current effects present in the FID. The existing DW-PRESS sequence would require a trigger event prior the FID acquisition and a careful temporal and spatial alignment between the FID from the spectroscopy voxel and the estimated eddy current effects from FIDs measured by the probes.

### 4.4 Gradient non-uniformities

Gradient coils are often designed with a limited field of view (FOV) to minimize nerve stimulation caused by rapidly changing magnetic fields over time. However, this design choice will increase gradient spatial non-uniformity, which becomes more noticeable as one moves away from the isocenter. In high-performance gradient systems like the one used here, gradient uniformity is often further compromized to achieve better performance. There is increased awareness of the importance of correcting for gradient deviations in diffusion MRI studies (Bammer et al., 2003; Guo et al., 2020; Mesri et al., 2020; Morez et al., 2021), but the specification of a gradient system is commercially sensitive information and not widely available.

Two strategies to represent the effective *b*-value were tested, i.e., incorporating the distribution of *b*-values, and representing that distribution using the median. Both strategies show similar deviations of the estimated ADC in DW-MRS, but both varied from the uncorrected nominal *b*-value, it is thus important to take into account the effective *b*-values when this information is available. Also, at lower gradient strength, and lower *b*-values—where the absolute signal change as a function of *b*-value is typically largest—gradient non-uniformities can bias quantitative estimates. In addition to *b*-value deviations, gradient non-uniformities will also result in distortions of the slice profile and thus voxel geometry. While the distortions we observed were small, it should be a consideration when placing voxels close to tissue boundaries, and conservative placement within the relevant tissue is advised, particularly when deviating far from the isocenter, where gradient non-uniformities are larger. Furthermore, there will likely be a small impact on chemical shift displacement error (CSDE). While CSDE is typically a linear chemical-shift-dependent translation of the effective voxel, when gradient non-uniformities are substantial, this effect is no longer a simple translation, and will result in metabolite-specific voxel deformation. This is an area that requires further study; however, we expect this effect to be minimal due to the lower amplitude of the imaging gradients, and the linear—rather than quadratic—effect that non-uniformities have on slice-selective gradients.

### 4.5 Table vibrations

The switching of strong diffusion gradients can cause mechanical vibrations at low frequencies within the scanner system and table. These vibrations could then be transmitted to the subject being scanned, resulting in artificial signal decay (Hiltunen et al., 2006) which artificially increases the measured ADC. While the Connectom scanner does, indeed, provide access to stronger gradients, it is not trivial how this may manifest as mechanical vibration. Interestingly, Setsompop et al. (2013) reported that the effects of acoustic noise generated by a 300 mT/m Connectom scanner were, in fact, lower than those at more conventional gradient strengths (40 mT/m). They postulated that the thicker, larger, and heavier gradient system of the Connectom scanner somewhat offset the larger amplitude of vibrational forces it generated (Setsompop et al., 2013).

Although the relatively lower directional variance of ADCs measured in the NIST phantom at the *G*_max_ = 300 mT/m setting was surprising, it is important to note that *in vivo* tissue stiffness and composition is different and likely more prone to vibration-induced artifacts. Indeed, our measurements in the CR show a particularly strong signal decay when diffusion-encoding is applied along z-direction. This might originate from tissue anisotropy and major white-matter tracts pointing along z (e.g., cortico-spinal or cortico-pontine tract) when diffusion of tNAA is considered. However, tCho shows consistent signal dropouts and diffusion in the low *b*-value range remains unaffected, which could point to effects related to table vibrations at high gradient amplitudes. Our hypothesis is that the CR—further away from the contact point between the head and the coil—is more strongly affected by random rotations induced by table vibrations than the OCC. This would be in line with previous observations of largest vibrations on the Connectom when diffusion-encoding is applied along the z-direction (Mueller et al., 2019). However, additional dedicated investigations with different sequence parameters (voxel position, diffusion-time) are required to elucidate this further.

In general, this artefact will be hardware- and sequence-specific but can be mitigated at the hardware level through careful consideration of the coil/gradient mounting (Ogura et al., 2006; Mueller et al., 2019), or even participant positioning, as different placement of padding around the head can lead to differing vibrational coupling between the brain and RF coil housing (Gallichan et al., 2010).

### 4.6 Other considerations

#### 4.6.1 Macromolecules

The macromolecular background is a potential confound to DW-MRS quantification (Ronen and Valette, 2007), with slowly-diffusing signal components persistent throughout the diffusion conditions. The impact of macromolecules is mitigated in this study as we acquired data at a longer TE, and parameterized the MM background during modeling. Experimentally acquired MM backgrounds are preferable—and can be acquired with ultra-strong gradient DW-MRS (Şimşek et al., 2022)—but are parameterization dependent, and are perhaps best-acquired at the cohort level in larger studies (Zöllner et al., 2023).

#### 4.6.2 Other sequences

While our study focused on a DW-PRESS implementation, the methodological considerations we identified are transferable to other DW-MRS localisation methods. The gradient non-uniformities can be corrected using the same principles, with simple modifications made to account for the specific gradient profile. Similarly, both of the eddy current correction methods considered here are also applicable to other single-shot localization schemes, and DW-PRESS might actually present a particularly challenging example. PRESS localization is known to be more susceptible to chemical shift displacement error than STEAM or sLASER, and this—coupled with the strong spatiotemporal dependence of eddy currents—can adversely affect the efficacy of the eddy-current correction in a metabolite-specific manner. The T_2_-based SNR gains provided by ultra-strong gradients are also transferable to other sequences with some caveats. Both DW-STEAM and DW-sLASER will benefit from the T_2_-based SNR gains, but are less sensitive to anomalous J-modulation effects than PRESS; however, our data suggest that this has a minimal effect on our DW-PRESS data. DW-STEAM also benefits from the decoupling of diffusion time from the TE, and this can be used to circumvent some of the T_2_ limitations to achieve high *b*-values. However, when short diffusion times are required—for example, to probe shorter length scales and/or minimize motion artefacts—ultra-strong gradients can still provide benefits. Furthermore, when measuring metabolite diffusion in small anatomical structures such as thalamus, hippocampus, brainstem, or spinal cord, higher gradient amplitudes combined with DW-STEAM or DW-sLASER localization can enhance an accurate localization (with a minimal CSDE) and should be prefered over DW-PRESS ^2^.

Another practical consideration that we didn't directly investigate in this work is the effect of concomitant gradient fields. These are nonlinear transverse components to an applied gradient field that arise from Maxwell's equations, and are typically more prominent at lower static field strengths and higher gradient field strengths (Baron et al., 2012). Our DW-PRESS sequence implementation utilized symmetric gradient timing to mitigate the impact of this effect, but for non-symmetric gradient implementations—for example, free gradient waveforms—this effect becomes an important consideration that should be corrected in a prospective manner (Baron et al., 2012; Szczepankiewicz et al., 2019).

#### 4.6.3 Other preprocessing steps

In this study, a retrospective outlier rejection method was used to identify and remove suspected motion-corrupted transients by comparing subsequent transients to the first. While 3 transients were removed in one such case—at the highest *b*-value—this did not impact the ADC estimations using the mono-exponential and kurtosis representations, which were performed using lower *b*-values. However, this may be adversely affect the SNR of highest *b*-value acquisitions in the other models. Weighting using the CRLBs mitigated the impact this had on model performance. Furthermore, if the initial transient of a given series is corrupted by motion, a high rate of outlier rejection would be observed. While this case wasn't encountered in these data, a secondary process—perhaps comparing to the median spectrum—would circumvent this issue. Our approach was conservative, and an alternative approach could be to monitor bulk motion in a prospective manner (Andrews-Shigaki et al., 2011; Saleh et al., 2020).

We have previously investigated the impact of phase and frequency correction methods on DW-MRS data (Jenkins, 2021), and the inherent SNR-dependent performance of such methods. Signal denoising techniques could mitigate this somewhat, and show promise for DW-MRS data (Mosso et al., 2022); however, care must be taken not to invalidate assumptions about noise characteristics during further modeling steps (Dziadosz et al., 2023).

#### 4.6.4 Modeling

While we focused on the practical considerations here, alternative DW-MRS modeling procedures could be beneficial, particularly leveraging high *b*-values and/or the higher SNR to further disentangle compartments (i.e., cylinders and spheres, Supplementary Figure S4, Supplementary Table S1). With the current data, sufficient support was not found for a more complex model, but future work could investigate this with specifically-acquired data.

Multi-spectrum modeling of MRS data—fitting the frequency and *b*-value dimensions in a single step, rather than independently—is another promising avenue which improves model parsimony and the stability of diffusion measures to experimental variation (Tal, 2023), and notably, allows stable parameterization of the macromolecules and baseline. Several groups have worked to develop this methodology in recent years e.g., FitAid (Chong et al., 2011; Adalid et al., 2017), FSL-MRS (Clarke et al., 2021), and Osprey (Oeltzschner et al., 2020).

### 4.7 Limitations

We were able to demonstrate the SNR improvement of incorporating ultra-strong gradients. However, cardiac and nerve stimulation limits place firm lower bounds on gradient ramp time and restrict the achievable gradient amplitudes at shorter TE. As a result, at TEs below 70 ms, the maximum gradient amplitude of the Connectom scanner is not achievable within the echo time, at least with the DW-PRESS sequence and diffusion gradient configuration we used. This limits the potential benefit provided by the ultra-strong gradient system. However, in practice, the ultra-high *b*-values we achieved in this study might not be necessary for all applications, and sacrificing diffusion weighting in favour of the improved SNR brought by lower TE might be preferable. This would be particularly beneficial when smaller voxel sizes or shorter scan times are a necessity. Moreover, for DW-PRESS, shorter TE also reduces the diffusion time, probing smaller length scales and different aspects of cell morphology. In practice, lower *b*-values and shorter TE might be preferable for detecting cell morphology changes in order to reduce scan time and/or voxel size.

Our relatively small cohort of volunteers limits the interpretability of the reported ADCs. A larger study is required to elucidate the effects of diffusion time, and systematically validate microstructure across tissue types. Moreover, while our multiple-gradient-condition acquisition allowed reasonable comparisons with conventional gradient systems, a systematic repeatability study using independent gradient systems would be necessary to fully vindicate these results. Indeed, the Connectom gradient design differs from that of a lower-gradient system, affecting gradient non-uniformity even at low gradient amplitudes, and cross-scanner comparisons would automatically include such differences (Gudino and Littin, 2023).

Furthermore, while our focus in this work was in the implementation of DW-MRS with ultra-strong gradients, and circumvention of major artefacts, we primarily limited our analysis to the major metabolites.

## 5 Conclusion

We successfully implemented acquisition and data processing strategies for ultra-strong gradient DW-MRS. We report diffusion coefficients which conform with the existing literature. Simulated SNR gains are experimentally confirmed, and results indicate that confounding effects of the strong gradient system can be ameliorated.

## Data availability statement

The datasets presented in this article are not readily available because we do not have ethical consent to make the *in-vivo* datasets acquired for this study publicly available. Requests to access the datasets should be directed to TaxC@cardiff.ac.uk.

## Ethics statement

The studies involving humans were approved by Cardiff University School of Psychology. The studies were conducted in accordance with the local legislation and institutional requirements. The participants provided their written informed consent to participate in this study.

## Author contributions

CD-J: Data curation, Formal analysis, Investigation, Methodology, Software, Visualization, Writing – original draft, Writing – review & editing. AD: Formal analysis, Investigation, Methodology, Software, Visualization, Writing – original draft, Writing – review & editing. FF: Methodology, Software, Writing – review & editing. EK: Formal analysis, Investigation, Methodology, Software, Visualization, Writing – original draft, Writing – review & editing. LM: Investigation, Methodology, Software, Writing – review & editing. CE: Conceptualization, Methodology, Supervision, Writing – review & editing. MA: Software, Writing – review & editing. DJ: Funding acquisition, Resources, Supervision, Writing – review & editing. IR: Conceptualization, Methodology, Software, Supervision, Writing – review & editing. FB: Conceptualization, Methodology, Software, Supervision, Writing – review & editing. CT: Conceptualization, Formal analysis, Investigation, Methodology, Software, Supervision, Visualization, Writing – original draft, Writing – review & editing.

## Footnotes

^1^Full code available https://github.com/CWDAVIESJENKINS/FIDA-Tarquin-Processing/tree/main/PipeLines/DWMRS.

^2^At ultra-high field (≥7T) PRESS localization is severely compromised by CSD, and STEAM or sLASER localization is recommended.

## Supplementary material

Supplementary Figure S1 Figure showing the MRS fitting residuals for the 300 mT/m **(left)** and 80 mT/m **(right)** acquisitions. Residuals are plotted in red, ascending in b-value from top to bottom.

Supplementary Figure S2 This figure shows the individual processed spectra (black) and fits (red) for the OCC **(left)** and CR **(right)** voxels for all b-values. Individual diffusion directions are grouped by diffusion value, and are ordered from top to bottom: x, y, and z, respectively.

Supplementary Figure S3 Eddy current phase evolution from three different gradient amplitudes: 295 mT/m (blue), 150 mT/m (red), and 50 mT/m (yellow).

Supplementary Figure S4 Fitting results applying an astrocylinder model and the estimated microstructural measures (free diffusivity D0, cylinder radius RC). Outliers were identified by the RMSE when iteratively removing a single data point from fitting.

Supplementary Figure S5 Fitting results applying a two-compartment model and the estimated microstructural measures (free diffusivity D0, sphere radius RS, cylinder radius RC). The fraction of cylinders fC was kept fixed at 0.8 and the standard deviation was estimated from bootstrapping. Outliers were identified by the RMSE when iteratively removing a single data point from fitting.

Supplementary Table S1 Listing of the *in vivo* results of microstructural measures (free diffusivity D0, sphere radius RS, cylinder radius RC) estimated from a two-compartment model. The double lined column delineation shows in the upper half the comparison of the 300 and 80 mT/m settings where diffusion-encoding was applied only along the z-direction, and in the lower half the comparison of the averaged diffusion metrics over three orthogonal diffusion-directions in the white matter rich corona radiata (CR) and grey matter rich occipital lobe (OCC) using the 300 mT/m setting. The fitting results can be found in Supplementary Figure S4. ^*^Fraction of cylinders fC was kept fixed at 0.8, c.f., text.

Supplementary Table S2 Full data quality information for the *in vivo* data. Each section contains entries for the Cramer-Rao lower bound (CRLB) of tNAA, tCho, and tCr, as well as the full width half maximum (FWHM), signal-to-noise ratio (SNR), points excluded by the motion-corruption metric (Excl.), and the mean frequency (Freq-Mean) and phase (Phs-Mean) shifts applied by spectral registration. Entries are ordered by increasing b-value per section.
