# Supplementary figures and images for: Practical considerations of diffusion-weighted MRS with ultra-strong diffusion gradients
Source: Front Neurosci. 2023 Dec 7;17:1258408. doi: 10.3389/fnins.2023.1258408 (PMC10740196; doi:10.3389/fnins.2023.1258408)

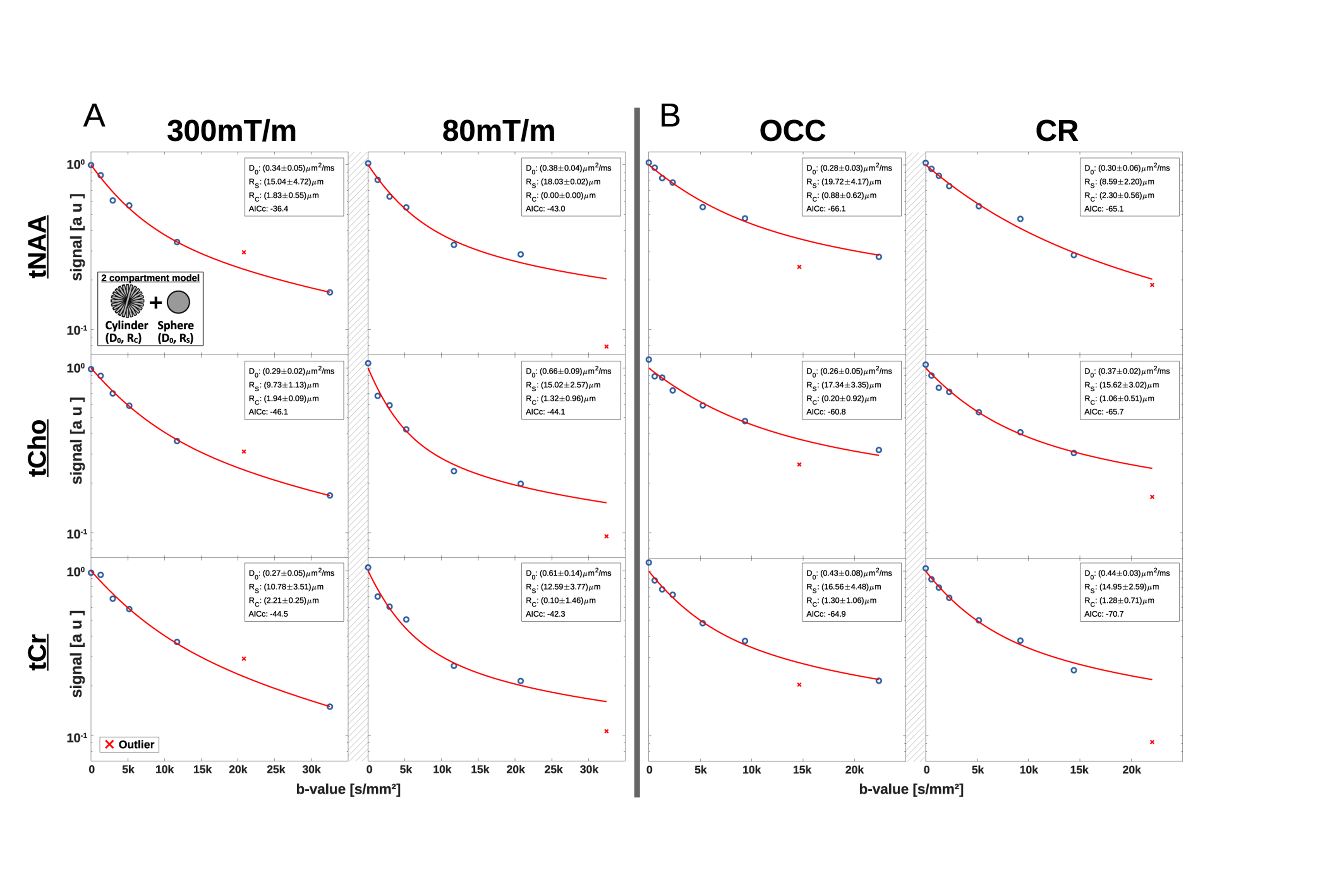

Supplement: Supplementary Figure S1 — Figure showing the MRS fitting residuals for the 300 mT/m (left) and 80 mT/m (right) acquisitions. Residuals are plotted in red, ascending in b-value from top to bottom. [file Image_1.tif]

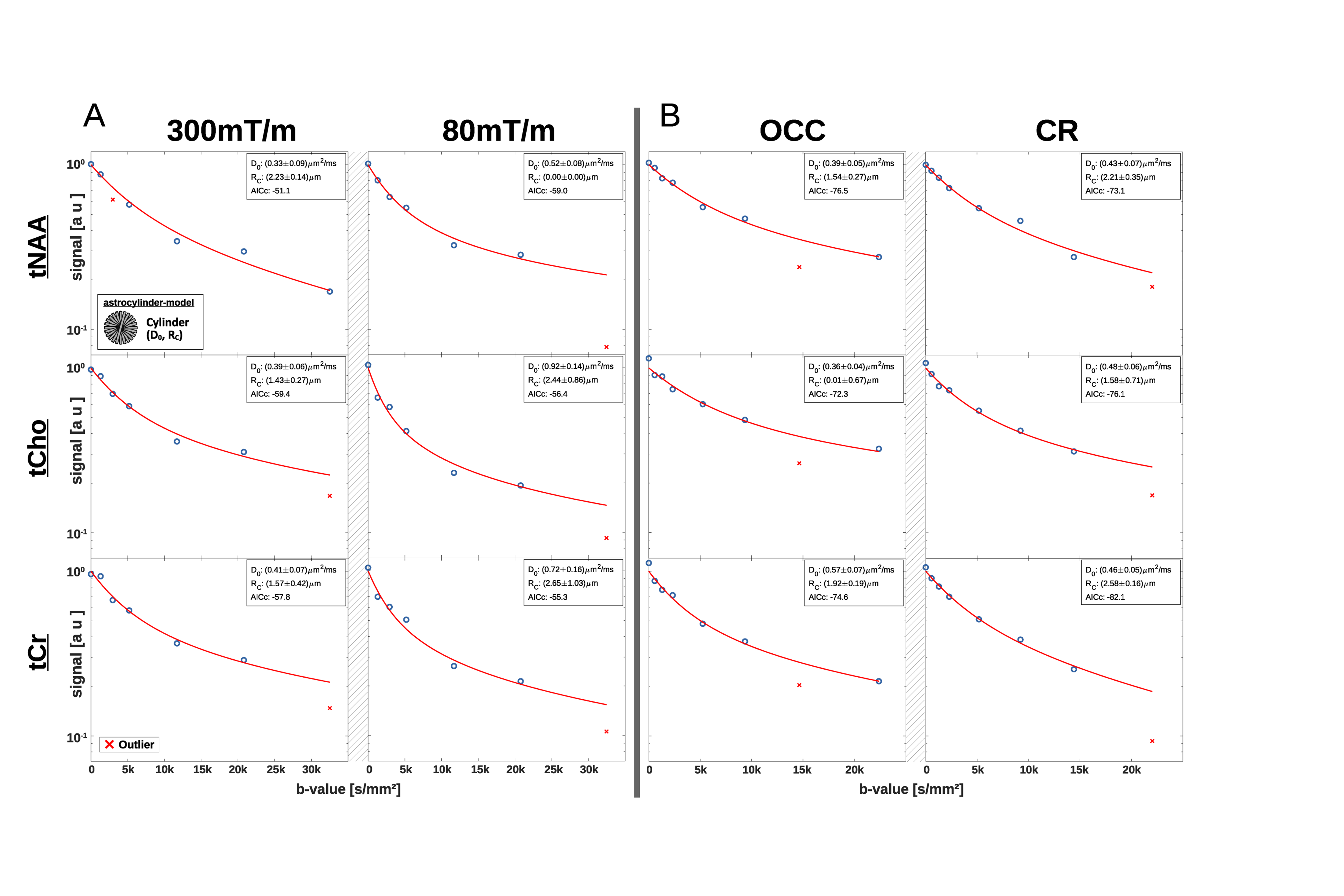

Supplement: Supplementary Figure S2 — This figure shows the individual processed spectra (black) and fits (red) for the OCC (left) and CR (right) voxels for all b-values. Individual diffusion directions are grouped by diffusion value, and are ordered from top to bottom: x, y, and z, respectively. [file Image_2.TIF]

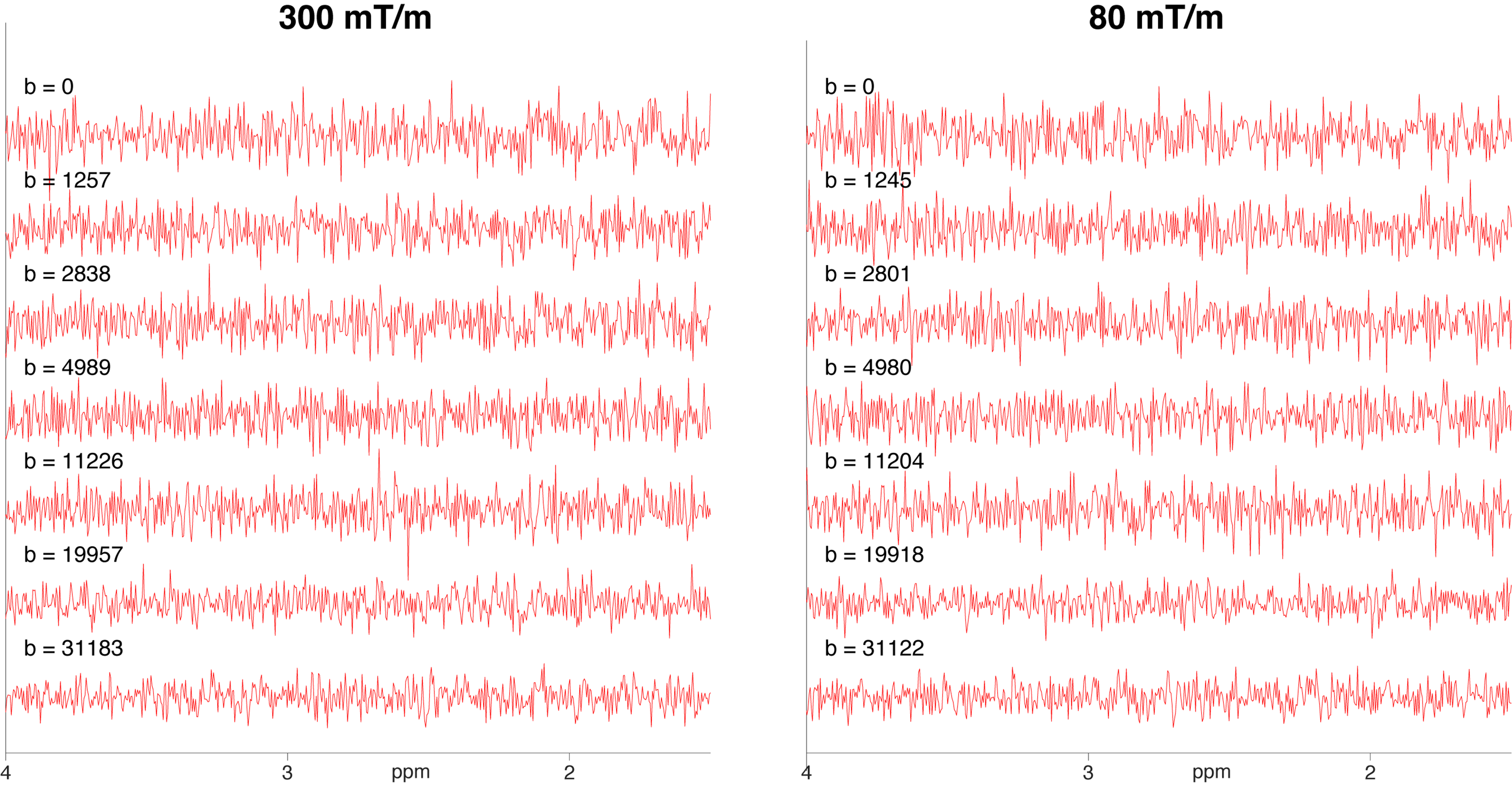

Supplement: Supplementary Figure S3 — Eddy current phase evolution from three different gradient amplitudes: 295 mT/m (blue), 150 mT/m (red), and 50 mT/m (yellow). [file Image_3.TIF]

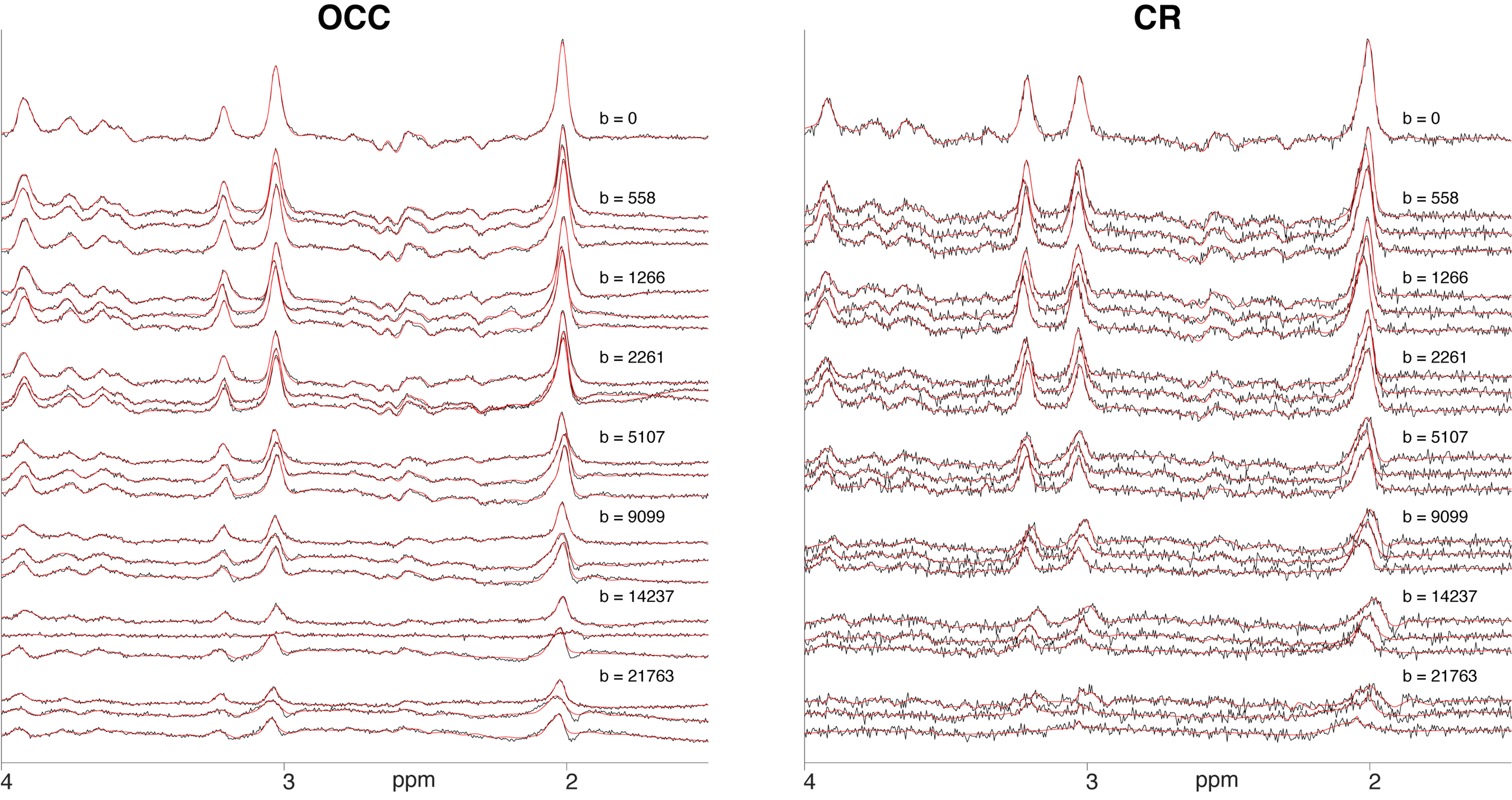

Supplement: Supplementary Figure S4 — Fitting results applying an astrocylinder model and the estimated microstructural measures (free diffusivity D0, cylinder radius RC). Outliers were identified by the RMSE when iteratively removing a single data point from fitting. [file Image_4.TIF]

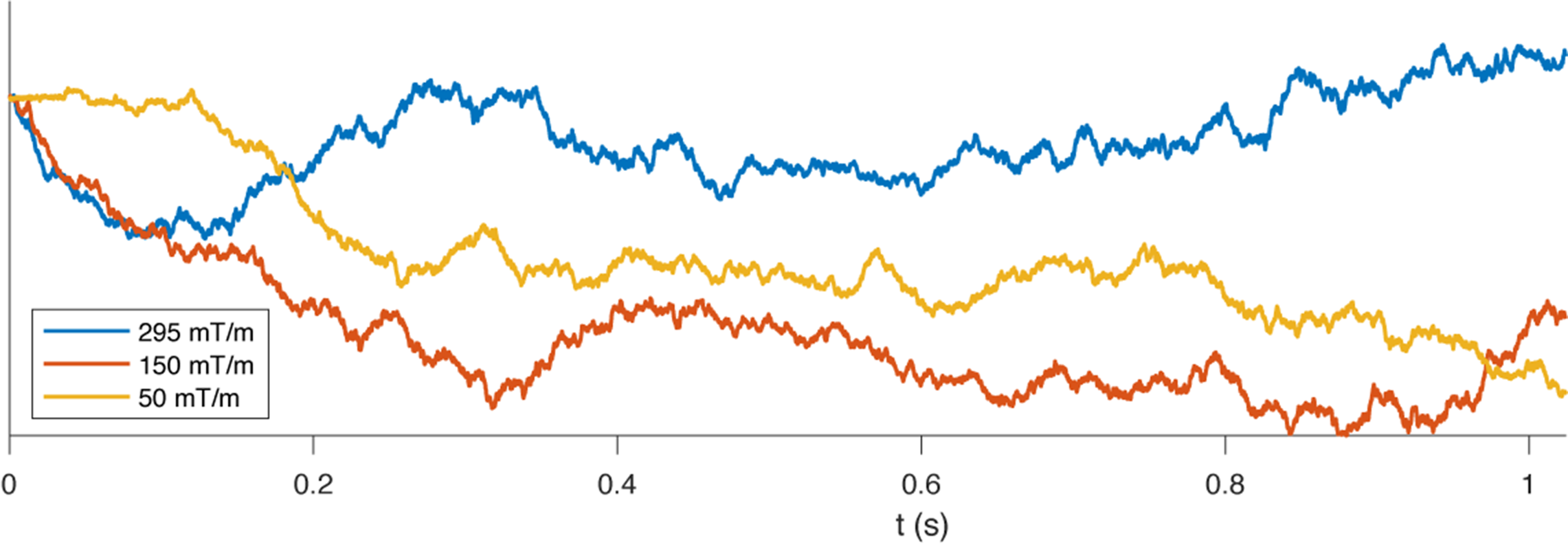

Supplement: Supplementary Figure S5 — Fitting results applying a two-compartment model and the estimated microstructural measures (free diffusivity D0, sphere radius RS, cylinder radius RC). The fraction of cylinders fC was kept fixed at 0.8 and the standard deviation was estimated from bootstrapping. Outliers were identified by the RMSE when iteratively removing a single data point from fitting. [file Image_5.TIF]

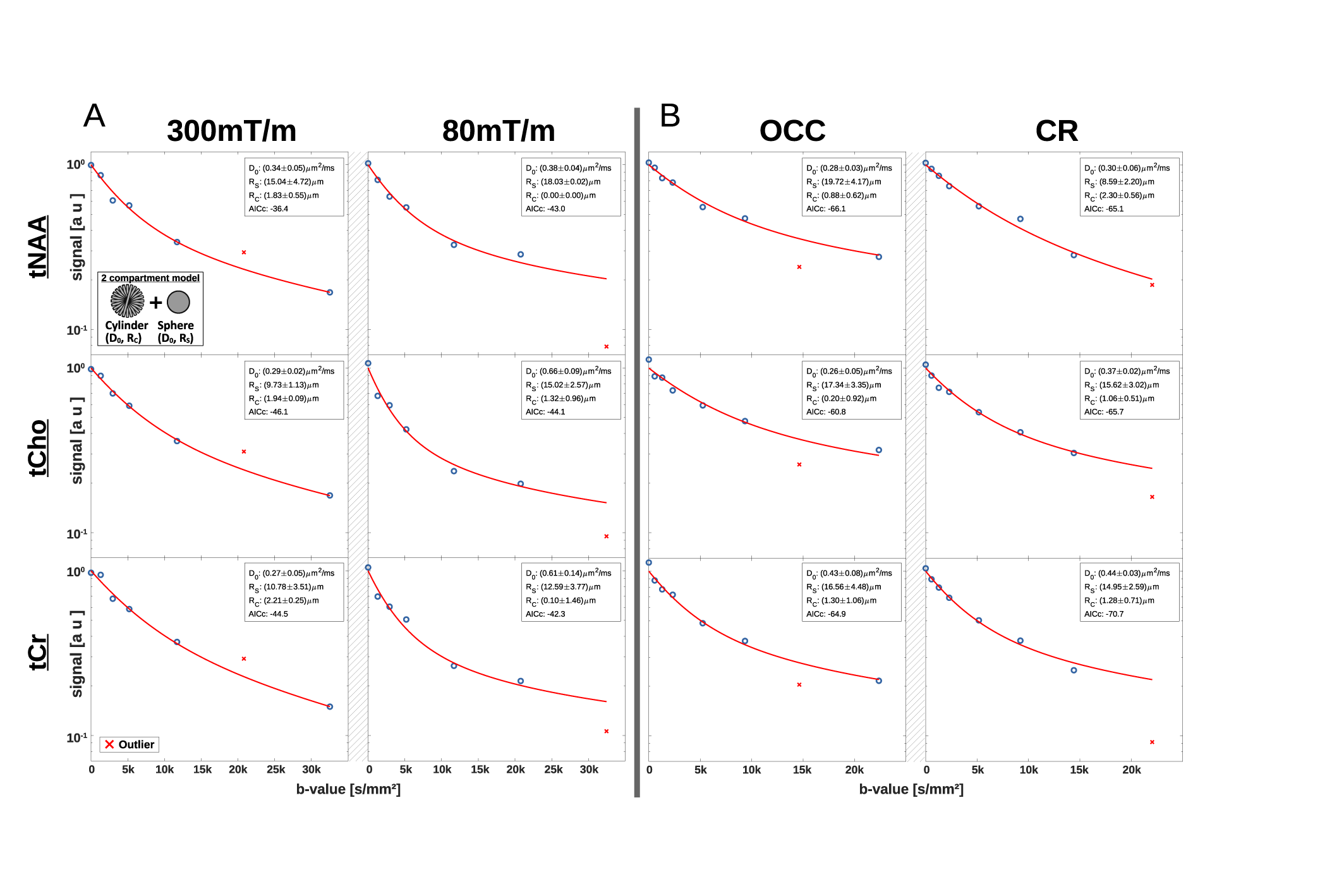

Supplement: Supplementary Table S1 — Listing of the in vivo results of microstructural measures (free diffusivity D0, sphere radius RS, cylinder radius RC) estimated from a two-compartment model. The double lined column delineation shows in the upper half the comparison of the 300 and 80 mT/m settings where diffusion-encoding was applied only along the z-direction, and in the lower half the comparison of the averaged diffusion metrics over three orthogonal diffusion-directions in the white matter rich corona radiata (CR) and grey matter rich occipital lobe (OCC) using the 300 mT/m setting. The fitting results can be found in Supplementary Figure S4. *Fraction of cylinders fC was kept fixed at 0.8, c.f., text. [file Data_Sheet_1.zip › SuppMat/figures_Sup/sup_2compModel_crop.tif]

# OCC

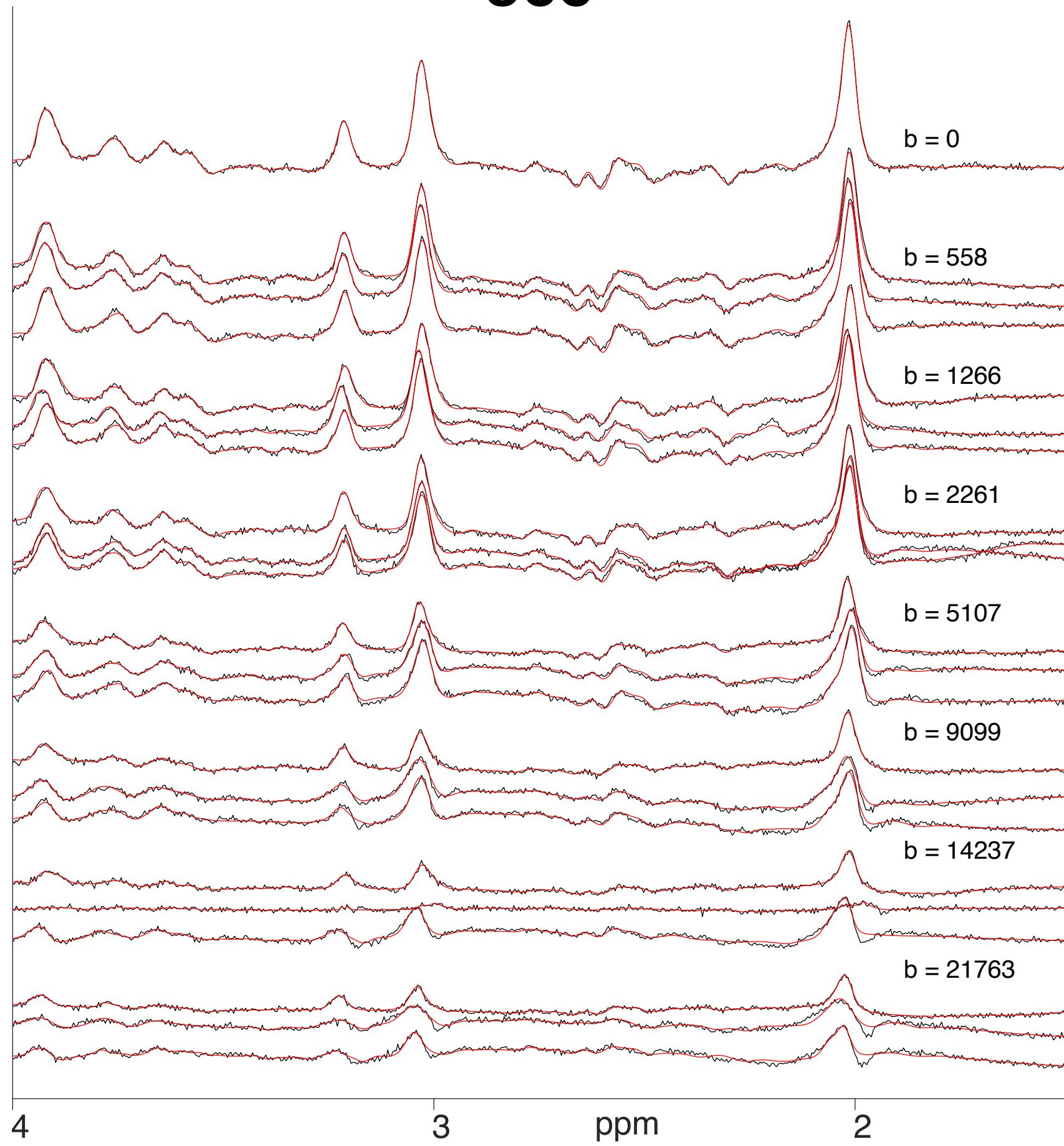

# CR

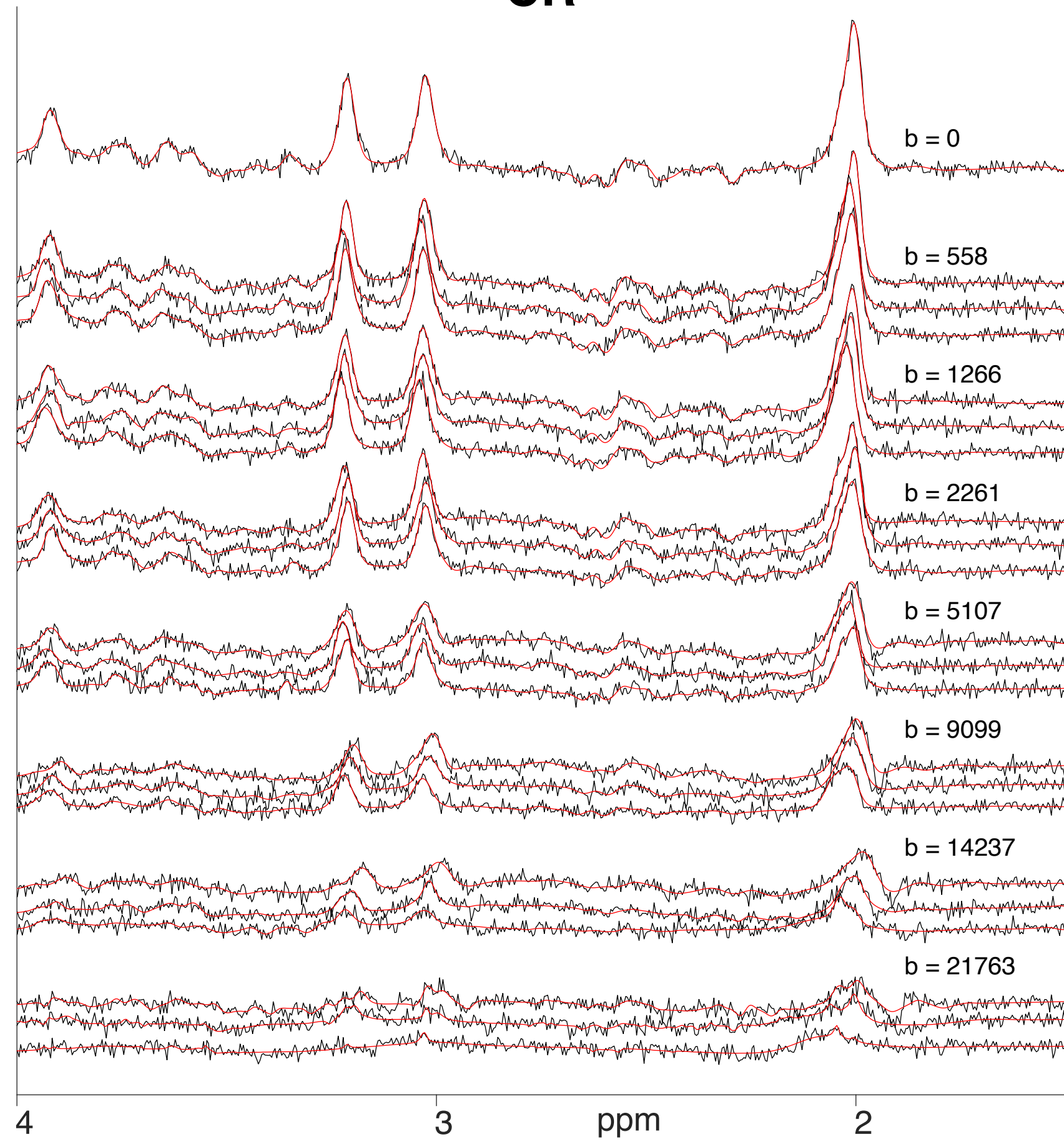

Supplement: Supplementary Table S1 — Listing of the in vivo results of microstructural measures (free diffusivity D0, sphere radius RS, cylinder radius RC) estimated from a two-compartment model. The double lined column delineation shows in the upper half the comparison of the 300 and 80 mT/m settings where diffusion-encoding was applied only along the z-direction, and in the lower half the comparison of the averaged diffusion metrics over three orthogonal diffusion-directions in the white matter rich corona radiata (CR) and grey matter rich occipital lobe (OCC) using the 300 mT/m setting. The fitting results can be found in Supplementary Figure S4. *Fraction of cylinders fC was kept fixed at 0.8, c.f., text. [file Data_Sheet_1.zip › SuppMat/figures_Sup/sup_GMWM_Specs.pdf]

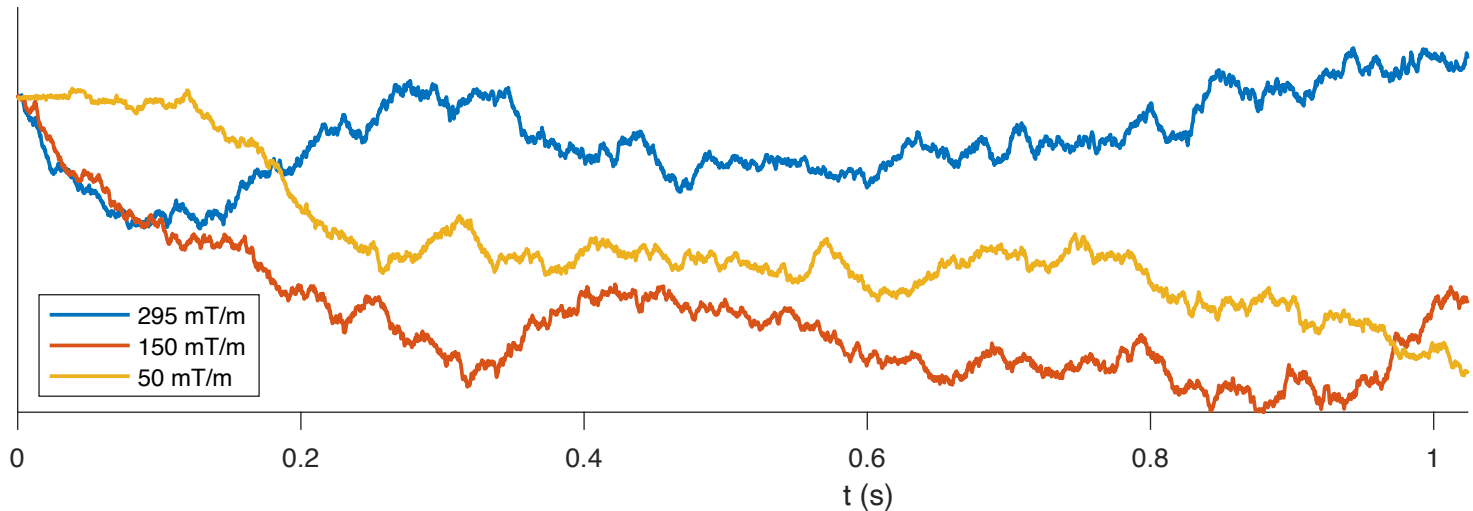

Supplement: Supplementary Table S1 — Listing of the in vivo results of microstructural measures (free diffusivity D0, sphere radius RS, cylinder radius RC) estimated from a two-compartment model. The double lined column delineation shows in the upper half the comparison of the 300 and 80 mT/m settings where diffusion-encoding was applied only along the z-direction, and in the lower half the comparison of the averaged diffusion metrics over three orthogonal diffusion-directions in the white matter rich corona radiata (CR) and grey matter rich occipital lobe (OCC) using the 300 mT/m setting. The fitting results can be found in Supplementary Figure S4. *Fraction of cylinders fC was kept fixed at 0.8, c.f., text. [file Data_Sheet_1.zip › SuppMat/figures_Sup/sup_ECCphase.pdf]

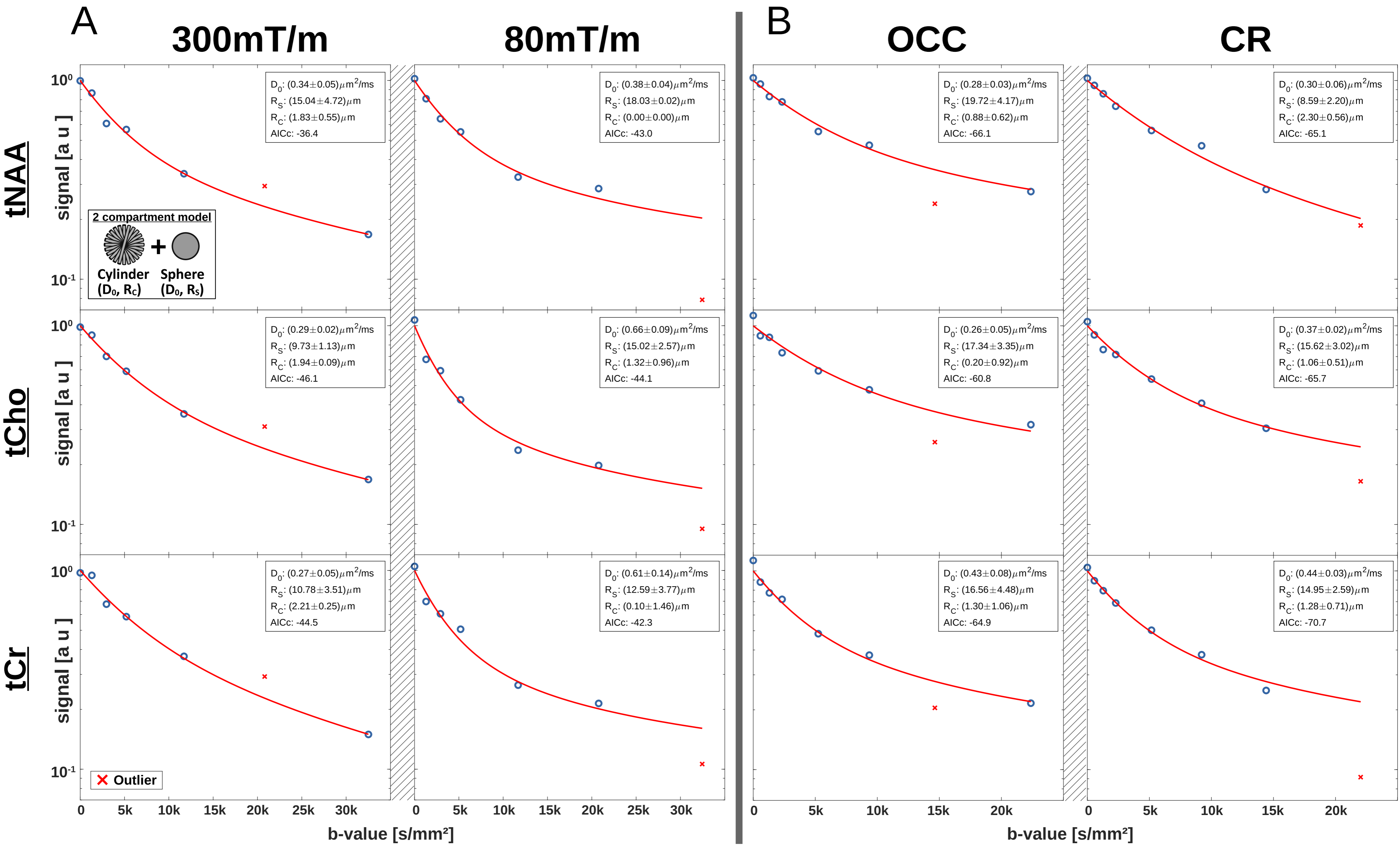

Supplement: Supplementary Table S1 — Listing of the in vivo results of microstructural measures (free diffusivity D0, sphere radius RS, cylinder radius RC) estimated from a two-compartment model. The double lined column delineation shows in the upper half the comparison of the 300 and 80 mT/m settings where diffusion-encoding was applied only along the z-direction, and in the lower half the comparison of the averaged diffusion metrics over three orthogonal diffusion-directions in the white matter rich corona radiata (CR) and grey matter rich occipital lobe (OCC) using the 300 mT/m setting. The fitting results can be found in Supplementary Figure S4. *Fraction of cylinders fC was kept fixed at 0.8, c.f., text. [file Data_Sheet_1.zip › SuppMat/figures_Sup/sup_2compModel_crop.pdf]

**300 mT/m**

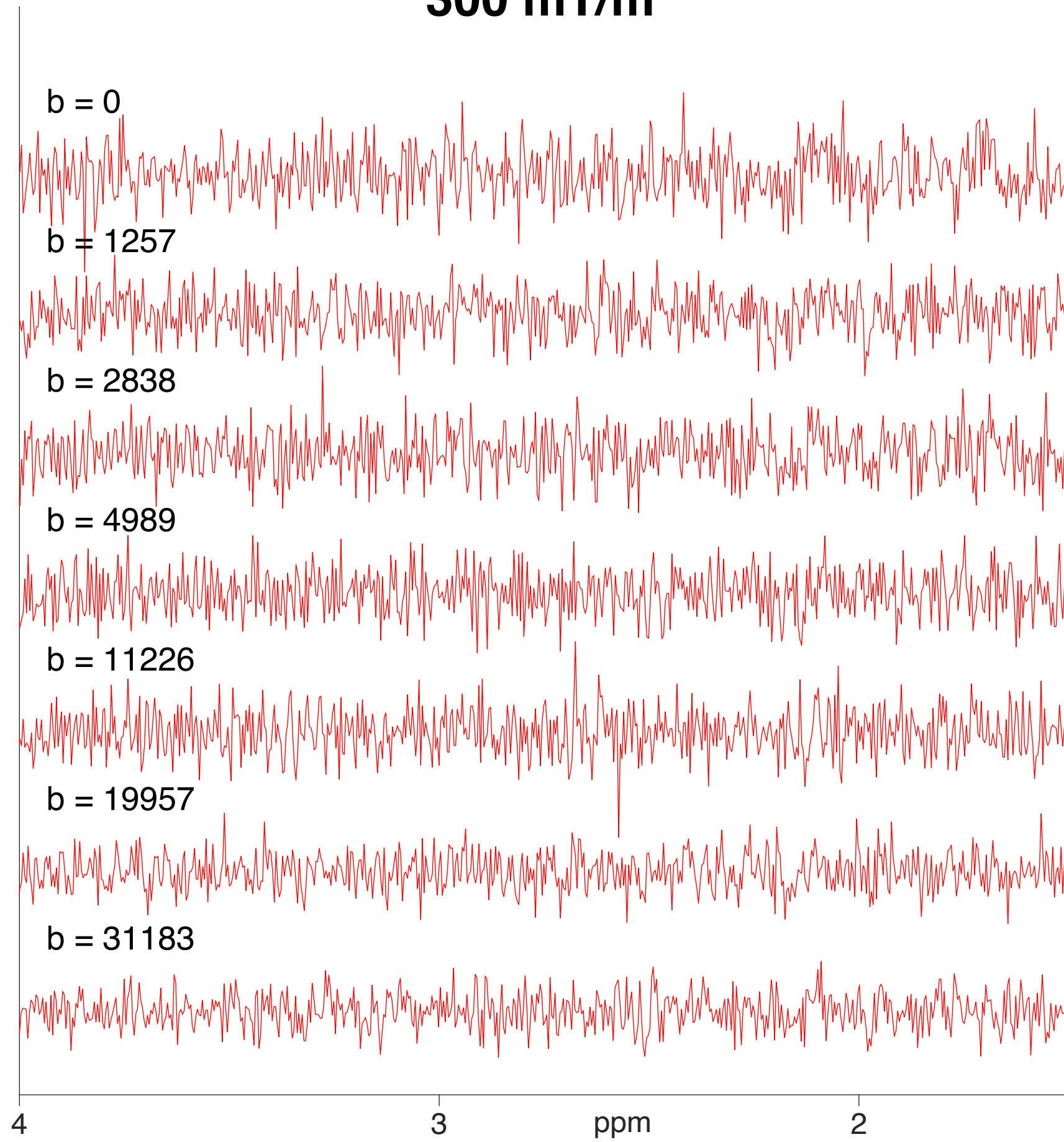

**80 mT/m**

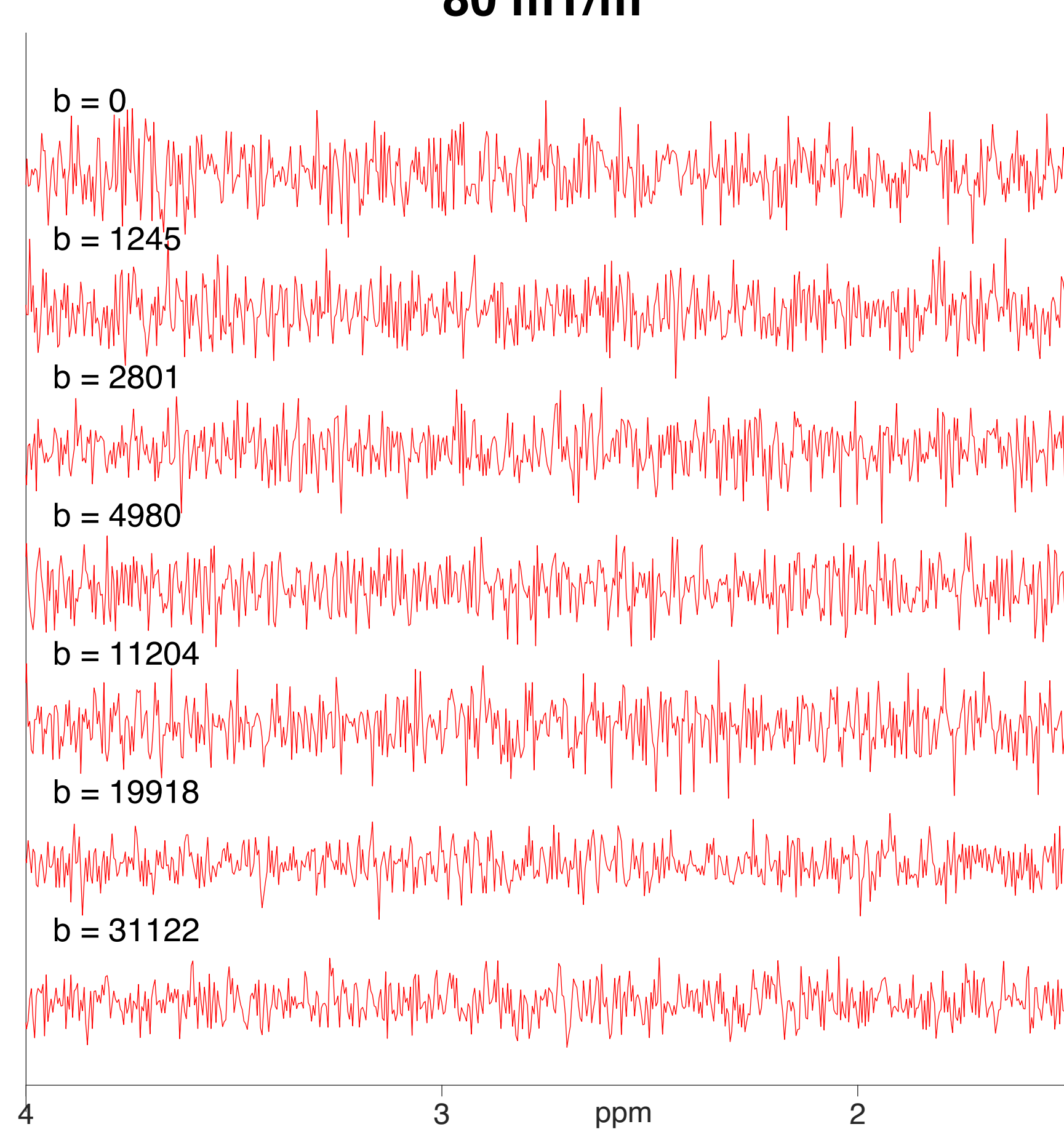

Supplement: Supplementary Table S1 — Listing of the in vivo results of microstructural measures (free diffusivity D0, sphere radius RS, cylinder radius RC) estimated from a two-compartment model. The double lined column delineation shows in the upper half the comparison of the 300 and 80 mT/m settings where diffusion-encoding was applied only along the z-direction, and in the lower half the comparison of the averaged diffusion metrics over three orthogonal diffusion-directions in the white matter rich corona radiata (CR) and grey matter rich occipital lobe (OCC) using the 300 mT/m setting. The fitting results can be found in Supplementary Figure S4. *Fraction of cylinders fC was kept fixed at 0.8, c.f., text. [file Data_Sheet_1.zip › SuppMat/figures_Sup/sup_ECC_residuals.pdf]

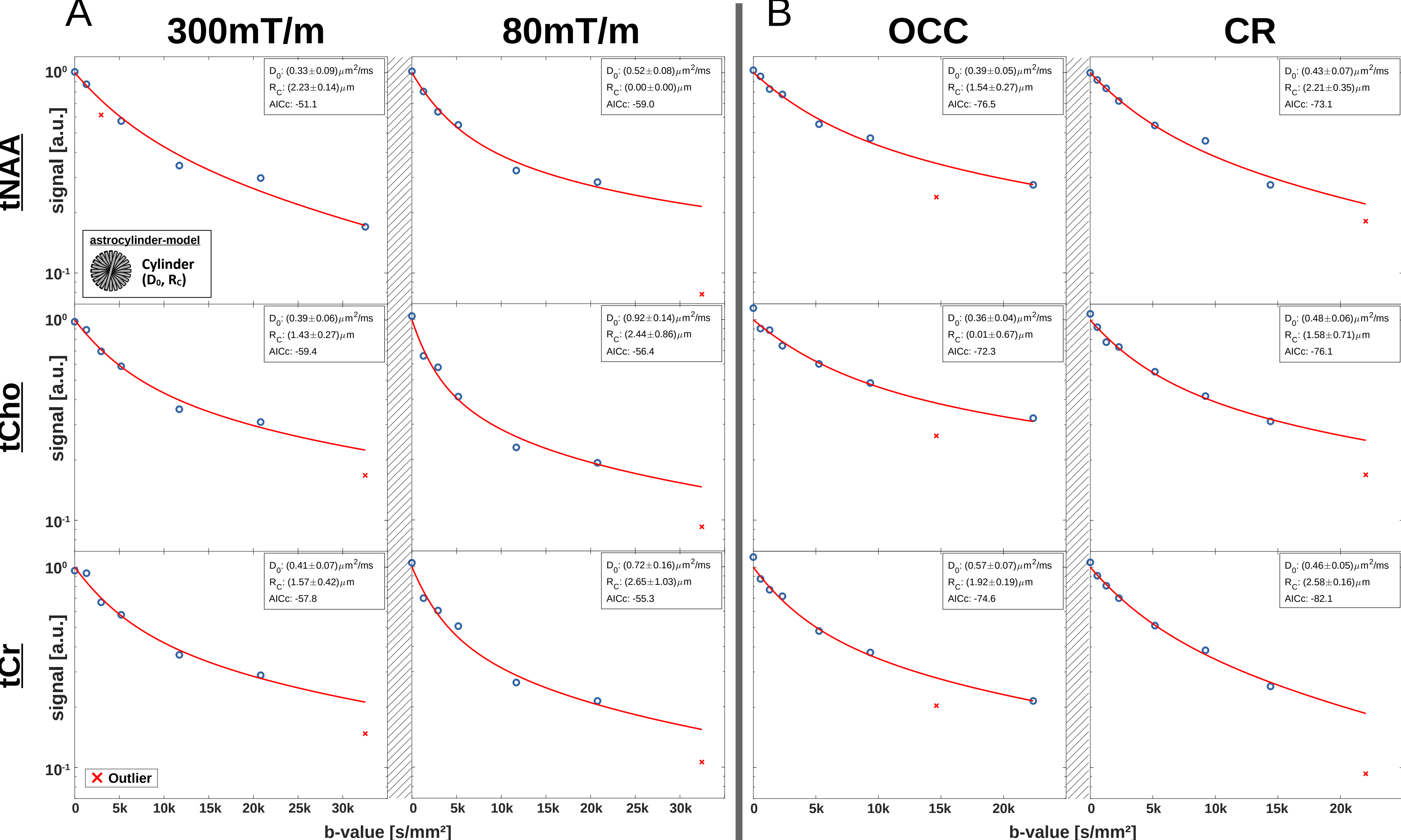

Supplement: Supplementary Table S1 — Listing of the in vivo results of microstructural measures (free diffusivity D0, sphere radius RS, cylinder radius RC) estimated from a two-compartment model. The double lined column delineation shows in the upper half the comparison of the 300 and 80 mT/m settings where diffusion-encoding was applied only along the z-direction, and in the lower half the comparison of the averaged diffusion metrics over three orthogonal diffusion-directions in the white matter rich corona radiata (CR) and grey matter rich occipital lobe (OCC) using the 300 mT/m setting. The fitting results can be found in Supplementary Figure S4. *Fraction of cylinders fC was kept fixed at 0.8, c.f., text. [file Data_Sheet_1.zip › SuppMat/figures_Sup/sup_astroCylModel_crop.pdf]
